# Supplementary material for: Rhomboid Family Pseudoproteases Use the ER Quality Control Machinery to Regulate Intercellular Signaling
Source: Cell. 2011 Apr 1;145(1):79–91. doi: 10.1016/j.cell.2011.02.047 (PMC3149277; doi:10.1016/j.cell.2011.02.047)
Supplement: Table S1. Genetic Interaction between Drosophila iRhom and Non-EGFR Signaling Pathways, Related to Figure 4 — A number of genes with known roles in developmental signaling were tested for their ability to interact genetically with homozygous iRhom mutants. iRhom homozygosity did not modify the phenotypes of indicated mutations (Mao and Freeman, 2009; Freeman and Bienz, 2001). [file mmc1.pdf]

Table S1. Genetic Interaction between *Drosophila* iRhom and Non-EGFR Signaling Pathways, Related to Figure 4

A number of genes with known roles in developmental signaling were tested for their ability to interact genetically with homozygous *iRhom* mutants. *iRhom* homozygosity did not modify the phenotypes of indicated mutations (Mao and Freeman, 2009; Freeman and Bienz, 2001).

Signalling pathway	Mutation tested	Genetic interaction
Wg	*F76e, $pygo^{S123}$, $\Delta axin^{P}$, ΔTCF^{2}, UAS-cadi,en-Gal4*	No
Notch	*Dl^{rev10}, GMR-Su(H)DN, N^{55e11}, $Hairless^{P141}$*	No
Hedgehog	*hh^{21}, ptc^{9}, UAS-Smo5A,C765-Gal4*	No
Dpp	*mad^{12}, dpp^{hr92}*	No
