# Supplementary figures and images for: Rhomboid Family Pseudoproteases Use the ER Quality Control Machinery to Regulate Intercellular Signaling
Source: Cell. 2011 Apr 1;145(1):79–91. doi: 10.1016/j.cell.2011.02.047 (PMC3149277; doi:10.1016/j.cell.2011.02.047)

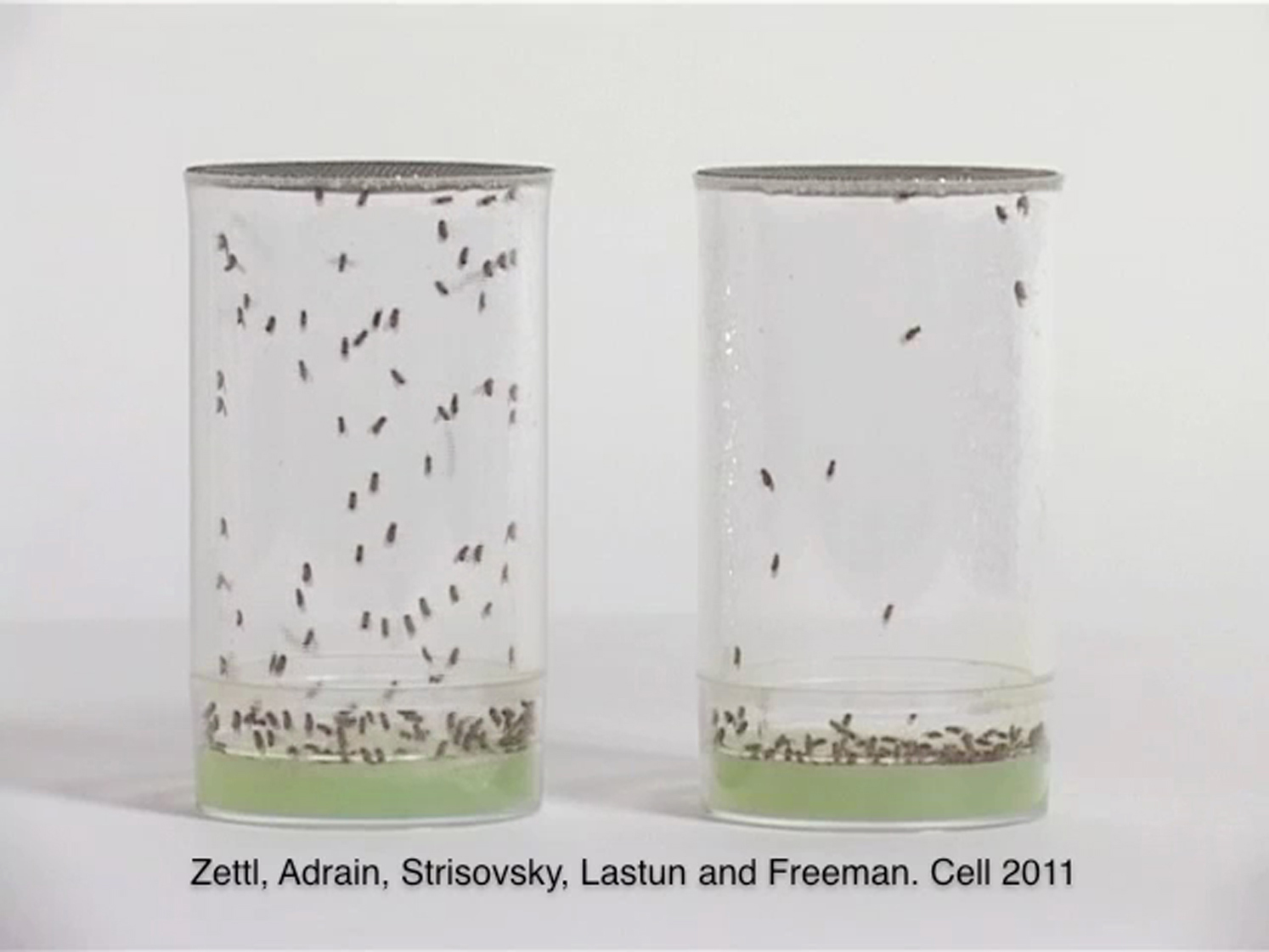

Supplement: Movie S1. iRhom Mutants Are Less Active and Responsive to Shaking Stimulus, Related to Figure 3 — In contrast to iRhom KO1/KO1 (on the right), iRhomKO1/+ flies (on the left) are highly active when the cages were banged on the table. An increased arousal threshold is one of the characteristics of sleep (Hendricks et al., 2000; Shaw et al., 2000). [file mmc2.jpg]
