# Supplementary material for: Rhomboid Family Pseudoproteases Use the ER Quality Control Machinery to Regulate Intercellular Signaling
Source: Cell. 2011 Apr 1;145(1):79–91. doi: 10.1016/j.cell.2011.02.047 (PMC3149277; doi:10.1016/j.cell.2011.02.047)
Supplement: Document S1. Article Plus Supplemental Information [file mmc3.pdf]

Cell

Rhomboid Family Pseudoproteases Use the ER Quality Control Machinery to Regulate Intercellular Signaling

Markus Zettl,[1] Colin Adrain,[1] Kvido Strisovsky,[1] Viorica Lastun,[1] and Matthew Freeman[1,*]
[1]MRC Laboratory of Molecular Biology, Hills Road, Cambridge CB2 0QH, UK
*Correspondence: mf1@mrc-lmb.cam.ac.uk
DOI 10.1016/j.cell.2011.02.047

SUMMARY

Intramembrane proteolysis governs many cellular control processes, but little is known about how intramembrane proteases are regulated. iRhoms are a conserved subfamily of proteins related to rhomboid intramembrane serine proteases that lack key catalytic residues. We have used a combination of genetics and cell biology to determine that these "pseudoproteases" inhibit rhomboid-dependent signaling by the epidermal growth factor receptor pathway in *Drosophila*, thereby regulating sleep. iRhoms prevent the cleavage of potential rhomboid substrates by promoting their destabilization by endoplasmic reticulum (ER)-associated degradation; this mechanism has been conserved in mammalian cells. The exploitation of the intrinsic quality control machinery of the ER represents a new mode of regulation of intercellular signaling. Inactive cognates of enzymes are common, but their functions are mostly unclear; our data indicate that pseudoenzymes can readily evolve into regulatory proteins, suggesting that this may be a significant evolutionary mechanism.

INTRODUCTION

Intramembrane proteases are widespread and control important cellular processes, but many questions remain about their function and regulation. There are four known families: presenilins/gamma secretase, implicated in Alzheimer's disease and Notch signaling (Haass and De Strooper, 1999); site-2 protease, first discovered as a regulator of cholesterol biosynthesis (Brown and Goldstein, 1997; Makinoshima and Glickman, 2006); signal peptide peptidase (Weihofen et al., 2002); and the most recently discovered, rhomboids (Urban et al., 2001). Rhomboids are serine proteases, conserved across all kingdoms of life, and they regulate processes as diverse as growth factor signaling, mitochondrial morphology, parasitic invasion, and bacterial protein translocation (Freeman, 2008; Urban, 2009). Extensive cell biology, biochemistry, and structural determination have provided insights into rhomboid mechanism, although, as with the other intramembrane proteases, much less is known about how rhomboid-dependent processes are regulated. This is a key question, as proteolysis is irreversible and often has potent cellular consequences.

Phylogenetic analysis of rhomboids identified a subgroup of rhomboid-like proteins that lack essential catalytic residues (Lemberg and Freeman, 2007). These "iRhoms" (for inactive rhomboids [Lemberg and Freeman, 2007]) are mysterious because, despite their predicted lack of protease activity, they are present in all sequenced metazoans, and their high degree of sequence identity implies selective pressure. Little is known about their function, but human iRhom1/Rhbdf1 has been reported to be necessary for the survival of some epithelial cancer cells (Yan et al., 2008) and may be linked to GPCR-mediated EGFR transactivation (Zou et al., 2009).

iRhoms exemplify a more general phenomenon: the existence of conserved but catalytically inactive cognates of enzymes. The widespread occurrence of this type of predicted protein has only been apparent since genome sequences have been available (Pils and Schultz, 2004; Bartlett et al., 2003), and their function is largely unknown. They are not generally encoded by pseudogenes and are therefore presumed to be expressed. Bioinformatic analysis has led to the suggestion that inactive enzyme cognates are disproportionately involved in regulatory processes (Pils and Schultz, 2004).

In *Drosophila*, active rhomboids are cardinal regulators of epidermal growth factor receptor (EGFR) signaling (Urban et al., 2001), and although it is unclear whether this activity is conserved in mammals (Blobel et al., 2009), recent evidence supports some evolutionary conservation (Adrain et al., 2011). Until now, nothing has been reported about iRhom function in *Drosophila*. We have used a combination of genetics and cell biology to discover that *Drosophila* iRhom regulates EGFR signaling. The EGFR pathway in *Drosophila* has become a model for understanding how signaling pathways are regulated with the precision that is necessary to control their multiple developmental and physiological roles (Shilo, 2003). *Drosophila* iRhom is expressed predominantly in neuronal cells, and its loss causes a "sleep"-like phenotype that is indistinguishable from gain-of-EGFR signaling in the central nervous system. Consistent with this, genetic interactions show that *Drosophila* iRhom counteracts the function of active rhomboids, specifically acting to inhibit EGFR signaling. As well as revealing the biological role of *Drosophila* iRhom, we have investigated the cellular

mechanism of iRhom function. Both fly and mammalian iRhoms, which are localized in the ER, inhibit secretion and intracellular levels of specific client proteins by promoting proteasomal degradation. Overall, these data imply that iRhoms regulate secretion of specific client proteins, which include the EGF family of growth factors. We show that they can target clients for proteasomal removal by ER-associated degradation (ERAD). In this way, fundamental cellular quality control machinery is exploited as a mechanism for regulating growth factor signaling.

RESULTS

iRhoms Lack Proteolytic Activity

Biochemical and structural analysis of rhomboid proteases has identified the two essential catalytic residues as being serine-201 and histidine-254 (numbered according to *E. coli* GlpG) (Wang et al., 2006). Phylogenetic alignment of eukaryotic rhomboid-like proteins identified a well-conserved subfamily in which one or both of these catalytic residues were missing, despite otherwise having clear sequence and topological similarity to the active rhomboids (Figure 1A) (Lemberg and Freeman, 2007). The human genes have been named Rhbdf1 and Rhbdf2, but the generic term iRhoms has been proposed to distinguish them from the active proteases (Lemberg and Freeman, 2007). The degree of conservation of these iRhoms, which are only found in metazoans, suggests that they are under evolutionary selective pressure, but as they are not predicted to be active proteases, their function is unclear. iRhoms have three prominent distinctions from active rhomboids: a much longer N-terminal cytoplasmic domain, a large and extremely conserved loop domain (the iRhom homology domain) between the first two transmembrane domains, and an invariant proline residue that is N terminal to the expected location of the catalytic serine (i.e., GPx replacing the GxS rhomboid catalytic motif). The significance of the iRhom-specific proline was investigated by generating variants of active rhomboids in which the residue immediately before the catalytic serine was mutated to proline. This mutation abolished the enzymatic activity of *Drosophila* Rhomboid-1 and strongly reduced mouse RHBDL-2 activity in a cell culture assay (Figures 1B–1D). The proline mutation also virtually abolished the in vitro activity of the purified bacterial rhomboid AarA (Strisovsky et al., 2009), demonstrating that it directly disrupts the enzyme (Figure 1E).

This prediction that iRhoms are themselves catalytically inert was tested by analyzing *Drosophila* iRhom, human iRhom1 (Rhbdf1), and mouse iRhom2 (Rhbdf2) in a cell culture proteolysis assay. No proteolytic activity was detected against the *Drosophila* EGFR ligands Gurken or Spitz, nor against mouse EGF (Figures 1B–1D), all substrates of multiple rhomboids (Urban et al., 2002a, 2002b; Adrain et al., 2011). We have further tested *Drosophila* and mammalian iRhoms against a variety of other rhomboid substrates without detecting any proteolytic activity (data not shown). Overall, these data support the idea that iRhoms have no proteolytic activity and demonstrate that their characteristic proline is sufficient to disrupt the rhomboid active site.

Drosophila iRhom Is Expressed in Neuronal Cells and Is Located in the ER

Unlike most sequenced metazoans, *Drosophila* has only a single iRhom, encoded by the *rhomboid-5* gene. In situ hybridization showed that *rhomboid-5/iRhom* RNA is expressed exclusively in the central nervous system of embryos: transcripts are strongly detected in the ventral nerve cord and brain (Figure 2A). In the third instar larva, *rhomboid-5/iRhom* expression was also largely restricted to neural tissue: specifically behind the morphogenetic furrow in the developing eye (Figure 2B), where the adult retina develops, and in the embryonic brain, with elevated levels in the optic lobes (Figure 2C). In adults, too, iRhom RNA is highly enriched in the nervous system and brain (http://www.flyatlas.org).

Individual active rhomboid proteases have specific locations in the Golgi apparatus and plasma membrane (Figure 2D) or in mitochondria (Lee et al., 2001; Urban et al., 2002a; McQuibban et al., 2003). We used COS7 cells to determine the localization of HA-tagged *Drosophila* iRhom, human iRhom1, and mouse iRhom2. All were located specifically in the endoplasmic reticulum (ER), as demonstrated by characteristic perinuclear and reticular staining and by colocalization with the ER marker protein disulphide isomerase (PDI) (Figure 2D). This is supported by an earlier observation that human iRhom1 is located in the ER in HNSCC 1483 cancer cells (Zou et al., 2009).

Drosophila iRhom Mutants Phenocopy Neuronal EGFR Activation

To investigate iRhom function, we used homologous recombination to generate a null mutant of *Drosophila iRhom* (Figure S1 available online). None of the three mutant lines that we isolated had discernible developmental defects. Homozygous mutations did, however, cause a severe decrease in the daytime activity pattern of adult flies compared to wild-type or heterozygous controls (Figure 3A and Movie S1). The specificity of this phenotype was confirmed by transheterozygotes between the targeted mutation and a pre-existing deletion for the region having the same phenotype (Figure 3A). Furthermore, *UAS-iRhom* expressed under the control of the neuron-specific *ELAV-Gal4* driver fully rescued the activity of the mutant flies (Figure 3B), whereas expression in muscle cells, under the control of *how24B-Gal4*, did not. This confirms the specificity of the phenotype and demonstrates that iRhom is required specifically in neurons, a result that is consistent with the nervous system-restricted expression pattern (Figures 2A–2C).

During the day, *iRhom* mutant flies sometimes do not move for periods of 1 hr, which is highly unusual. Prolonged periods of inactivity could, in principle, be caused by increased resting (a sleep-like state) or an impaired ability to move. To distinguish these two possibilities, we measured the activity of flies during periods of wakefulness. During their relatively few active periods (147 over the course of the experiment, compared to 820 in the control), mutants showed no reduction of movement; in fact, in those periods, they moved slightly more than wild-type or heterozygous controls (Figure 3C). To confirm this, we analyzed video recordings and found mutants and wild-type to have the same walking speed (Figure 3D). This indicates that *iRhom* mutant flies can move normally and implies that their overall

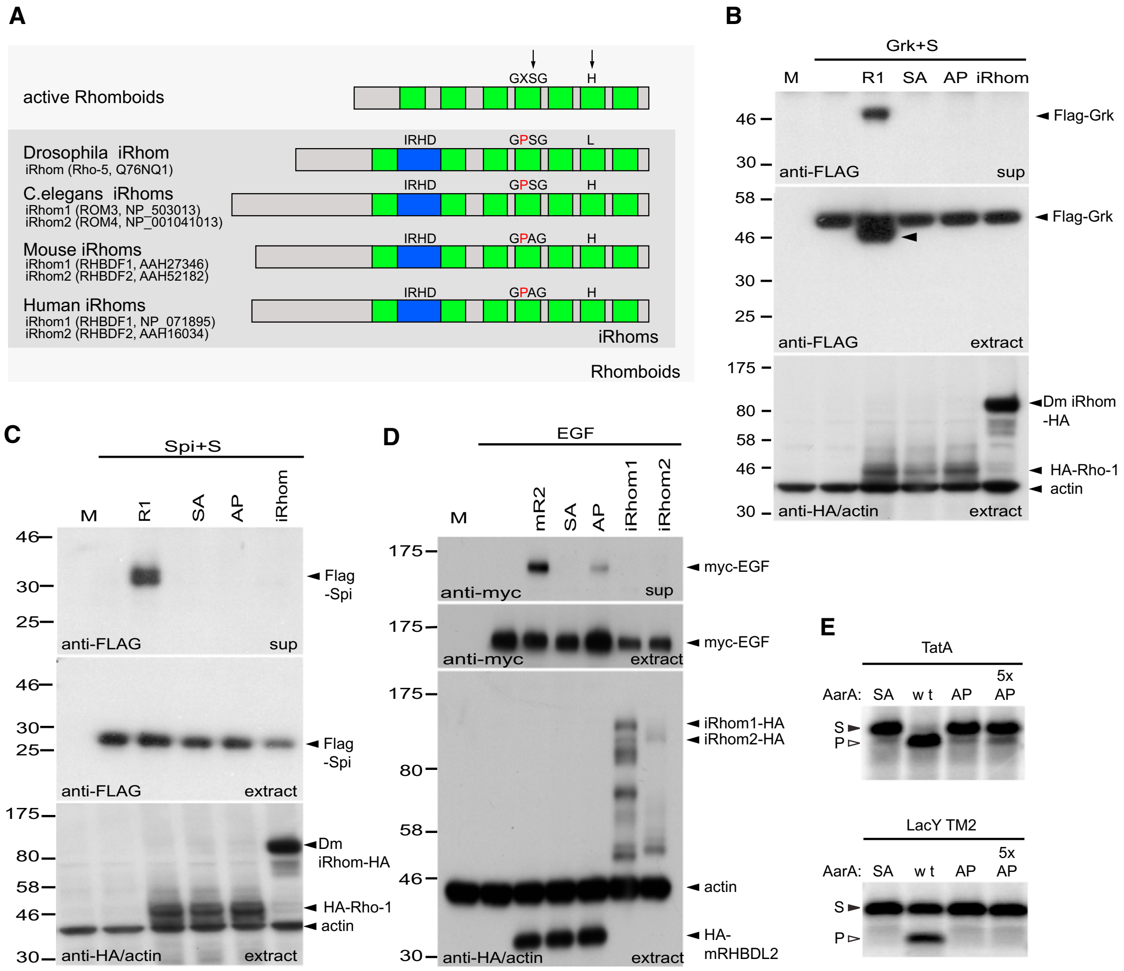

Figure 1. iRhoms Are Inactive Members of the Rhomboid-like Family

(A) iRhoms are highly conserved and lack the catalytic serine and/or histidine (arrows) of active rhomboids. Distinctive iRhom elements are long N termini, a conserved iRhom homology domain (IRHD, blue), and an invariant proline residue (red) before the catalytic serine. Protein names and identifiers are indicated. Note that the gene for *Drosophila* iRhom (Swiss-Prot:Q76NQ1; Rhomboid-5) is ambiguously predicted (Lemberg and Freeman, 2007).

(B and C) Western blots assaying proteolytic activity in COS7 cells. Flag-tagged Gurken and Star (Grk+S) (B) or Flag-Spitz and Star (Spi+S) (C) were cotransfected with HA-tagged versions of Rhomboid-1 (R1), a catalytic serine-to-alanine mutant of Rhomboid-1 (SA), a mutant Rhomboid-1 with an iRhom-like proline (AP), or *Drosophila* iRhom. Cleavage of substrate inside the cells (extract) and secreted into the supernatant (sup) was detected with anti-Flag antibody. Cell extracts were also probed with anti-HA and anti-actin as controls for rhomboid expression and gel loading. Wild-type Rhomboid-1 cleaves and releases Spitz and Gurken; the SA mutant, AP mutant, and iRhom show no activity. Once cleaved, Spitz is rapidly secreted, explaining the absence of a cleavage product in the cell extract (Urban and Freeman, 2003).

(D) A similar experiment using mouse HA-tagged RHBDL2 (mR2) and myc-tagged mouse EGF. The alanine-to-proline mutation (AP) strongly reduced EGF secretion, and neither human iRhom1 nor mouse iRhom2 showed any catalytic activity. Mammalian iRhoms have been reported to be proteolytically processed (Nakagawa et al., 2005), explaining the multiple bands in cell extracts. This assay was done in the presence of 10 μM BB94 to inhibit metalloprotease shedding. Throughout the paper, “M” represents a mock transfection control and “SA” represents a serine-to-alanine catalytic mutant.

(E) The iRhom-like proline mutation in the active site of a bacterial rhomboid protease abolishes its enzymatic activity. AarA (WT), its catalytic serine-to-alanine mutant (S150A, SA), and iRhom-like mutant (A149P, AP) were overexpressed in *E. coli* and purified in the presence of detergent. The substrates TatA and LacY TM2 were in vitro translated and radiolabeled. Enzyme and substrates were incubated at 37° for 40 min. Wild-type AarA concentrations were 280 nM for TatA and 560 nM for LacY TM2. Mutants were equimolar to the wild-type or 5-fold higher when indicated (5×). Cleavage products (P) were separated from the substrates (S) by SDS PAGE and detected by autoradiography.

inactivity is due to an increase in periods of inactivity between active episodes. Consistent with this, sleep, typically defined as periods of inactivity greater than 5 min (Greenspan et al., 2001), was significantly increased in *iRhom* mutants (Figure 3E).

This same inactivity phenotype is caused by activation of EGFR signaling upon transient CNS expression of Rhomboid-1 and Star (Foltenyi et al., 2007). This is significant because of the well-established functional relationship between active rhomboids and EGFR activation (Wasserman et al., 2000; Urban et al., 2001). Our results are therefore consistent with loss of *Drosophila* iRhom in the nervous system leading to activation of EGFR signaling, thereby decreasing daytime activity. This

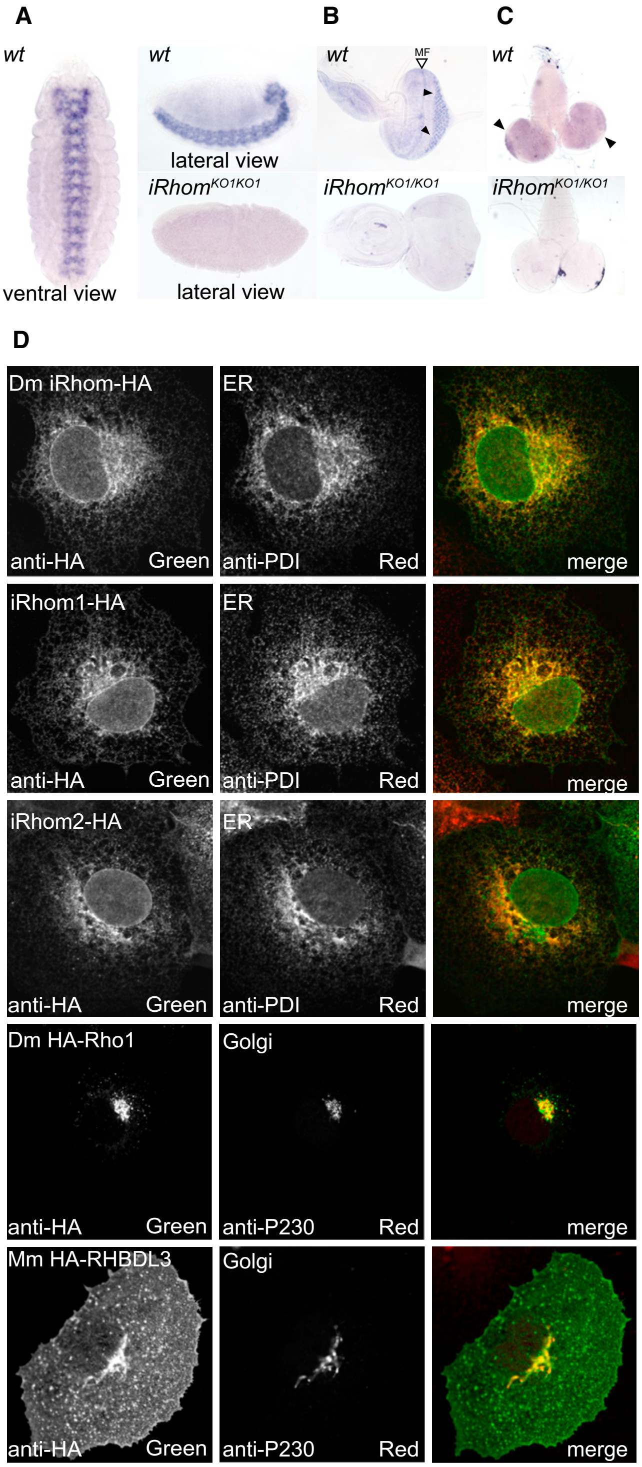

Figure 2. Expression and Cellular Location of iRhoms

(A–C) RNA in situ hybridization of wild-type and *iRhom* mutant *Drosophila* embryos, third-instar eye discs, and larval brains with a probe against *iRhom*.

(A) *iRhom* is expressed in the embryonic CNS, including the ventral nerve cord and brain.

(B) Third-instar larval eye discs showed staining posterior to the morphogenetic furrow (MF).

(C) *iRhom* expression is detected at low levels throughout the larval brain and is elevated in the optic lobes (arrowheads).

(D) HA-tagged *Drosophila* iRhom, human iRhom1, and mouse iRhom2 were located in the endoplasmic reticulum (ER) in COS7 cells. *Drosophila* Rho-1 localized to the Golgi apparatus, and the mouse rhomboid RHBDL3 localized to the Golgi apparatus, the plasma membrane, and punctate endosome-like structures. HA antibodies were used to detect iRhom; antiprotein disulfide isomerase (PDI) was an ER marker; and anti-P230 was used as a Golgi marker. See also Figure S1.

would imply that, under wild-type conditions, iRhom promotes wakefulness by inhibiting EGFR activity.

Drosophila iRhom Is a Specific Inhibitor of EGFR Signaling

The cellular mechanism by which EGFR signaling in the CNS regulates *Drosophila* sleep patterns is not yet understood, so to investigate further the genetic relationship between iRhoms and EGFR, we used the well-characterized systems of imaginal disc development. We looked for genetic interactions to test the hypothesis that iRhoms act as physiological inhibitors of EGFR activity. Halving the dose of iRhom, which alone caused no phenotype, strongly enhanced the rough eye caused by EGFR hyperactivity induced by Rhomboid-1 overexpression (Figure 4A). Conversely, removal of iRhom suppressed the eye phenotype caused by reduction of EGFR signaling, for example when Argos, Sprouty, or a dominant-negative form of the EGFR was overexpressed in the eye (Figure 4B). Both of these synergistic interactions imply that iRhom inhibits EGFR signaling; this was directly demonstrated by iRhom overexpression (using GMR-Gal4) suppressing the rough eye caused by the overexpression of active Rhomboid-1 (Figure 4C).

In another set of experiments, we examined the consequences of *iRhom* heterozygosity in combination with reduction in the known EGFR inhibitory molecules Sprouty and Argos. Removing one copy of *sprouty* triggered a mild extra wing vein phenotype in 6.4% of adult flies grown at 29°C; this was enhanced to 74.5% when the dose of *argos* was simultaneously halved (Figure 4D). Similarly, halving *iRhom* strongly enhanced the *sprouty* heterozygous wing phenotype: 62.5% of wings had mild, and 3.65% had strong extra vein phenotypes (Figure 4D). The additive effect of all three genes was further demonstrated in the triple-heterozygote combination, wherein mild (+) and severe (++) extra vein phenotypes were further increased to 87.5% and 10.3%, respectively (Figure 4D). Together, all of these synergistic genetic interactions indicate that iRhom acts in the same pathway as Argos and Sprouty and is an inhibitor of EGFR signaling.

Importantly, we also tested whether other developmentally significant signaling pathways were similarly perturbed by changes in the dose of iRhom. In an extensive series of experiments, we found no evidence for genetic interactions with Wg, Notch, Hedgehog, or Dpp signaling (Table S1). Overall, we conclude that *Drosophila* iRhom inhibits EGFR activity in vivo; that this role is particularly prominent in the nervous system but can be detected in the developing wing and eye under sensitized conditions; and that, although we cannot rule out a role in pathways that have not been tested, this effect is largely specific to the EGFR-signaling pathway.

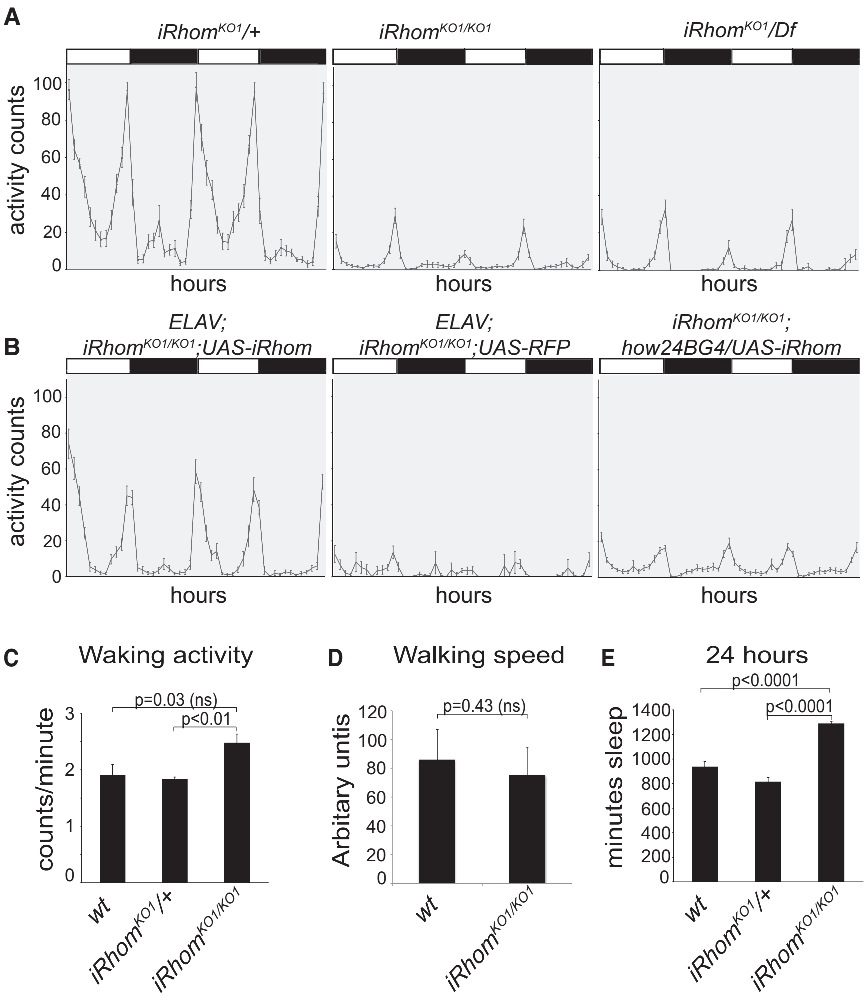

Figure 3. Loss of iRhom Results in Increased Sleep

(A and B) Activity levels over 2 days of male flies of indicated genotypes plotted as activity counts per hr (error bars represent mean ± SEM). Bars above the diagrams indicate light (white) and dark (black) periods of 12 hr each. Data represent measurements from two to four independent experiments.

(A) Activity patterns of heterozygous (*iRhom*^{KO1}/+; n = 18), homozygous *iRhom* mutant (*iRhom*$^{KO1/KO1}$; n = 16), and transheterozygotes (*iRhom*^{KO1}/*Df* (2L) J17; n = 17). *iRhom* homozygotes and transheterozygotes between *iRhom*^{KO1} and *Df(2L)J17* show highly suppressed activity levels.

(B) Neuronal expression of *UAS-iRhom* (n = 27), but not *UAS-RFP* (n = 24), by *ELAV-Gal4* rescues the inactivity phenotype of *iRhom*$^{KO1/KO1}$ (n = 16) flies. Expression of iRhom in muscle under the control of *how24B-Gal4* (n = 38) does not rescue the activity pattern.

(C) Waking activity, expressed as an average number of beam crossings in each minute in which activity was detected, was slightly increased in *iRhom*$^{KO1/KO1}$ (n = 17) flies compared to *iRhom*^{KO1}/+ (n = 16) and wild-type controls (n = 21). In this and subsequent panels, significance was determined with Student's t test (ns, not significant). Error bars represent mean ± SEM.

(D) The movement of wild-type (n = 5) and *iRhom*$^{KO1/KO1}$ (n = 5) flies was filmed. Fly movement was tracked during an active period, and the average walking speed (excluding short stops) was calculated in arbitrary units. Error bars represent mean ± SEM.

(E) Sleep, defined as periods of inactivity greater to or equal than 5 min, was significantly elevated in *iRhom*$^{KO1/KO1}$ flies (n = 39) compared to heterozygous (*iRhom*^{KO1}/+, n = 33) and wild-type (n = 44) controls. Error bars represent mean ± SEM.

See also Movie S1.

To confirm that disruption of the EGFR pathway also can explain the sleep-like iRhom phenotype, we looked for similar genetic interactions affecting activity (Figure 4E). Reduction of EGFR signaling by RNAi knockdown of the EGF receptor itself, or its activating ligand Spitz, significantly rescued the iRhom loss-of-function phenotype, supporting the idea that the lethargy in the mutant flies is indeed caused by excess EGFR activity (Figure 4E).

Drosophila and Mammalian iRhoms Specifically Inhibit Rhomboid-Triggered Ligand Release

The genetic data described above demonstrate a physiological role for *Drosophila* iRhom but cannot provide direct mechanistic insight. For this, we turned to cell culture, asking whether iRhoms can interfere with the function of active rhomboid proteases. When Gurken, a *Drosophila* EGF family ligand, and *Drosophila* Rhomboid-1 are coexpressed in COS7 cells, Gurken is cleaved and its extracellular domain is released into the culture medium (Figure 5A) (unlike Spitz, Gurken does not require the action of Star [Yogev et al., 2008]). Coexpression of *Drosophila* iRhom inhibited the release of soluble Gurken induced by Rhomboid-1 (Figure 5A). This effect was specific, to the extent that the release into the medium of two other proteins, Delta, a transmembrane protein subject to metalloprotease shedding, and Wnt3A, a secreted protein, was unaffected by iRhom expression (Figure 5B and Figure S2A). We also ruled out a different kind of nonspecific effect by coexpressing with Gurken and Rhomboid-1 similar levels of an unrelated polytopic ER protein, Unc93B (Brinkmann et al., 2007). This caused no reduction of Gurken release (Figure 5C), implying that the iRhom effect was not a nonspecific consequence of overexpressing a polytopic membrane protein (for example, caused by overload of the ER). We also found that the release of Spitz, another *Drosophila* EGFR ligand, is similarly blocked by iRhom coexpression (data not shown). Together, these results show that *Drosophila* iRhom specifically inhibits EGFR ligand release from cells. Because iRhoms are conserved in all metazoans, we asked whether their function might be conserved between *Drosophila* and mammals. When human iRhom1 (data not shown) or mouse iRhom2 were coexpressed with mouse EGF and the mouse active rhomboid, RHBDL2 (Adrain et al., 2011), secretion of soluble EGF was inhibited (Figure 5D), implying

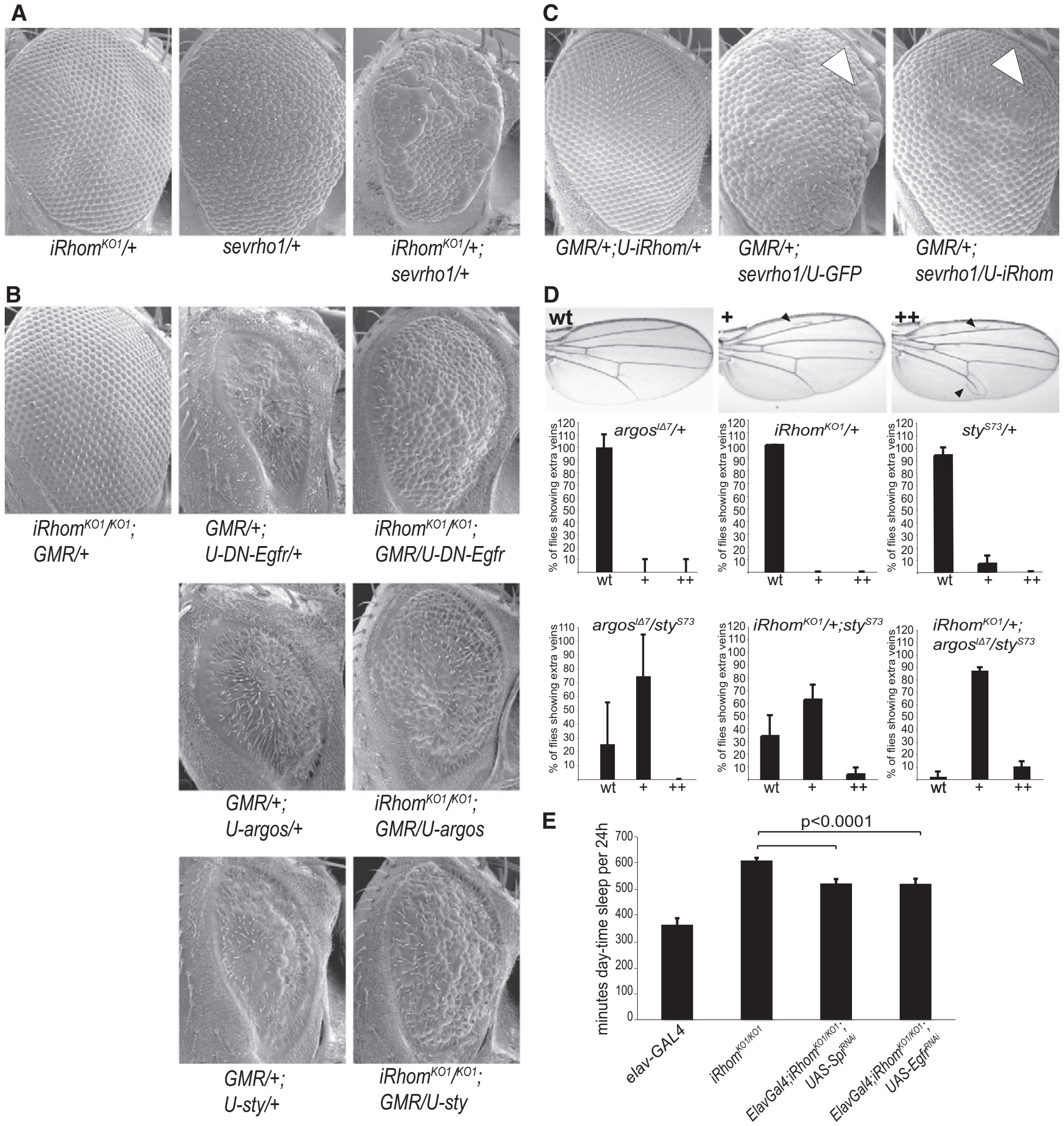

Figure 4. *Drosophila* iRhom Inhibits EGFR Signaling

(A) The rough eye caused by sevenless-driven Rhomboid-1 overexpression (*sev-rho-1*/+) is strongly enhanced by halving the dose of iRhom (*iRhom*^{KO1}/+; *sev-rho-1*/+).

(B) Loss of iRhom suppressed the rough eyes caused by reduction of EGFR signaling mediated by UAS-driven: *dominant negative Egfr* (*U-DN-Egfr*); *argos* (*U-argos*); and *sprouty* (*U-sty*). The driver in all cases was the eye-specific *GMR-Gal4*.

(C) Expression of *UAS-iRhom* under the control of *GMR-Gal4* suppressed the rough eye caused by *sev-rhomboid-1* expression. This was particularly obvious at the anterior edge of the eye indicated by white arrowheads.

(D) Genetic interactions in the wing. Examples of wing phenotypes and severity levels indicated by wild-type (WT), mild (+), and severe (++) are shown. Wings of flies that were heterozygous for *argos* (*argos*$^{l\Delta7}$/+) or *iRhom* (*iRhom*^{KO1}/+) were wild-type; 6.4% (± 6.4%) of wings that were heterozygous for *sprouty* (*sty*^{S73}/+) showed mild extra vein phenotypes (black arrowheads), typical of slight EGFR hyperactivation. A transheterozygous combination of *sty*^{S73} and *argos*$^{l\Delta7}$ enhanced the extra vein phenotype (74.5% ± 6.4% with mild extra veins). Similarly, halving *iRhom* in combination with *sty*^{S73} (*iRhom*^{KO1}/+; *sty*^{S73}/+) enhanced the phenotype (62.5% ± 11.6% mild; 3.65% ± 5.4% severe). *iRhom*^{KO1}/+; *sty*^{S73}/ *argos*$^{l\Delta7}$ flies showed a further increase in penetrance and strength of the extra vein phenotype (87.5% ± 2.3% mild; 10.3% ± 3.8% severe). All flies for this experiment were grown at 29°C. Error bars represent standard deviations of three to four independent experiments (n = 50 per experiment).

(E) *iRhom*$^{KO1/KO1}$ flies (n = 44) showed increased daytime sleep compared to controls (*elav-GAL4*, n = 37). Inhibition of EGFR signaling in the nervous system by expression of RNAi constructs against *spitz* (*elav-GAL4; iRhom*$^{KO1/KO1}$*; UAS-spi*^{RNAi}, n = 27) and *Egfr* (*elav-GAL4; iRhom*$^{KO1/KO1}$*; UAS-Egfr*^{RNAi}, n = 36) significantly ($p < 0.0001$) suppressed the *iRhom*$^{KO1/KO1}$ sleep phenotype. Error bars represent mean ± SEM.

See also Table S1.

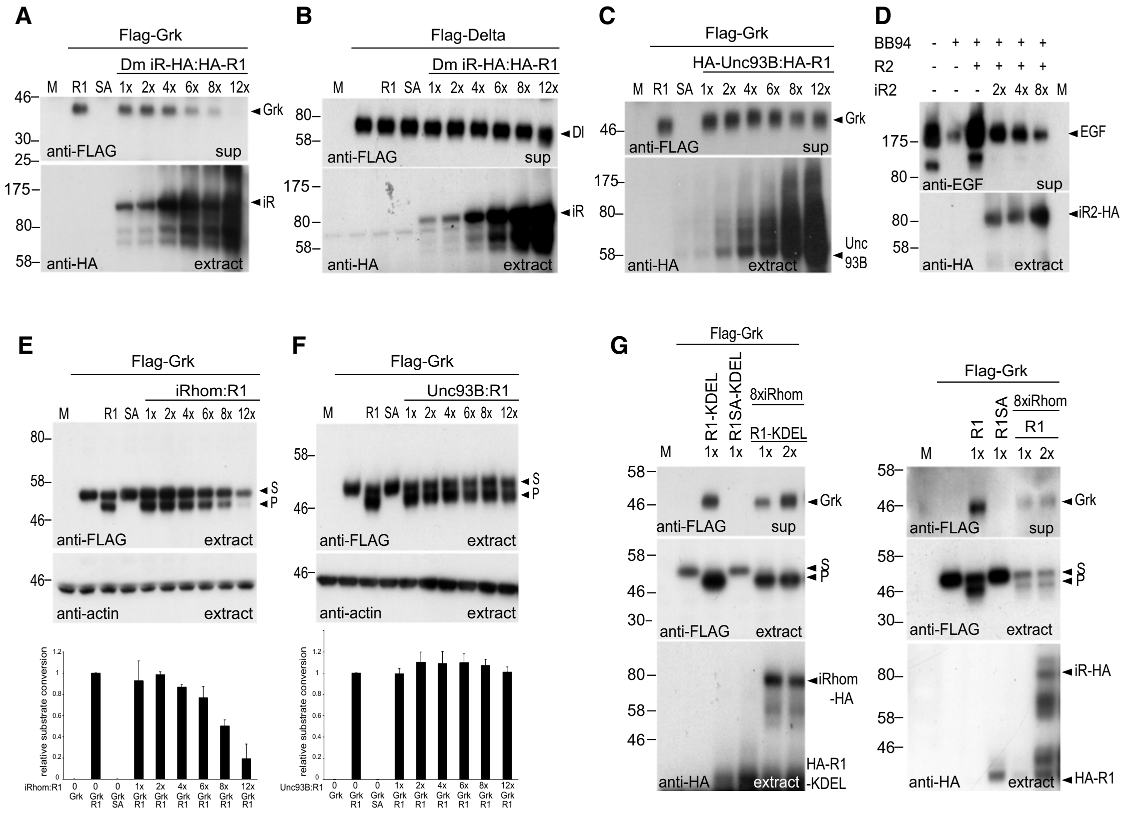

Figure 5. iRhom Inhibits Secretion of EGF Family Ligands

(A–C) COS7 cell supernatants (sup) were analyzed for Grk secretion, and cell extracts were blotted for levels of HA-tagged iRhom and Unc93B (Brinkmann et al., 2007). The ratios of iRhom to *Drosophila* Rhomboid-1 (R1) indicate relative amounts of transfected DNA.

(A) Rhomboid-1 (R1)-induced secretion of Flag-Grk was inhibited by increasing amounts of iRhom.

(B) Secretion of the FLAG-tagged Delta was unaffected by increasing amounts of iRhom.

(C) Overexpression of the ER resident polytopic membrane protein Unc93B did not interfere with Grk secretion.

(D) Mouse iRhom2 (iR2) inhibited mouse RHBDL2 (R2)-induced secretion of EGF. Secreted mouse EGF was detected by anti-EGF. The metalloprotease inhibitor BB94 suppressed nonspecific shedding of EGF. Increasing amounts of HA-tagged mouse iRhom2 (bottom) inhibited RHBDL2-mediated EGF secretion (top).

(E and F) *Drosophila* iRhom destabilizes intracellular Gurken. Rhomboid-1-mediated Grk processing was inhibited by iRhom (E), but not Unc93B (F). Histograms show relative substrate (S) to product (P) conversion from the western blots above. The iRhom-to-Rho1 (iRhom:R1) and Unc93B:R1 ratios indicate relative amounts of transfected DNA. Equal loading was confirmed by probing cell extracts for actin levels. Error bars represent mean ± SD.

(G) The iRhom effect was rescued by expression of ER-localized, but not Golgi-localized, active rhomboid. KDEL-tagged Rhomboid-1 (R1-KDEL), but not the inactive serine-to-alanine mutant Rhomboid-1 (Rho1 SA-KDEL), induced secretion of Grk in supernatants. A constant amount of iRhom inhibited Grk secretion (8× more iRhom DNA transfected than R1-KDEL). Increasing amounts of HA-tagged R1-KDEL rescued Grk secretion. Using the same approach, untagged Rhomboid-1, which is Golgi localized (see Figure 2D), did not rescue Grk secretion (right). HA-tagged iRhom, R1-KDEL, and R1 were detected in cell extracts. Flag-tagged Grk was detected in the supernatants (top) and extracts (middle). The Grk substrate band was absent when R1-KDEL is used because of efficient processing when substrate and enzyme are both in the ER.

See also Figure S2.

that both mammalian iRhoms had a similar function to their *Drosophila* counterpart.

iRhoms Specifically Reduce Intracellular Levels of EGF Family Ligands

The specific inhibitory effect of iRhom on the accumulation of soluble Gurken in the cell culture medium was also reflected in cell extracts, but in this case, we detected two distinct effects. First, iRhom coexpression reduced Gurken cleavage by Rhomboid-1, expressed as relative substrate conversion (Figure 5E). Second, the total level of intracellular Gurken, intact and cleaved, was reduced by iRhom coexpression (Figure 5E). Again, controls imply that this was a specific effect: the polytopic ER protein Unc93B did not reduce Gurken cleavage or intracellular levels (Figure 5F), and neither Wnt3A nor Delta were destabilized by iRhom expression (Figures S2B and S2C). Finally, all of these effects were efficiently rescued by the additional expression of an ER-targeted version of Rhomboid-1, but only slightly rescued by high levels of wild-type Rhomboid-1, which is located in the Golgi apparatus (Figure 5G). This result is important for a number of reasons. First, it further demonstrates that the iRhom effect is not caused by a nonspecific disruption of the secretory pathway.

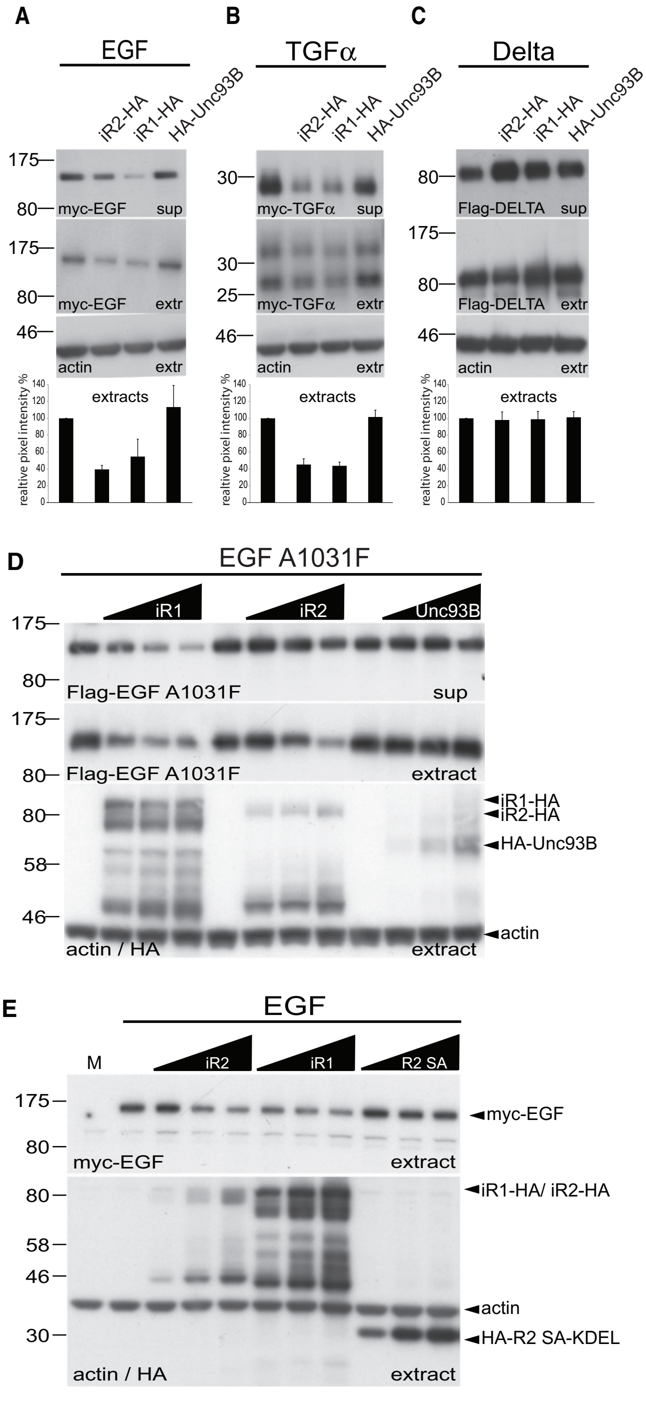

Figure 6. iRhoms Induce Degradation of EGFR Ligands Independently of Active Rhomboids

(A and B) Secretion and intracellular levels of the indicated EGFR ligands were decreased by human iRhom1 (iR1-HA) and mouse iRhom2 (iR2-HA) but unaffected by mouse Unc93B.

(C) Secretion and intracellular levels of Flag-tagged Delta were unaffected by iR1, iR2, and Unc93B. EGFR ligands and Delta were detected with myc and Flag antibodies, respectively. The effects of mammalian iRhoms and Unc93B on intracellular levels of EGF, TGFα, and Delta were quantified from three independent experiments and plotted as relative percentages (error bars indicate standard deviations). Levels of iRhom1, iRhom2, and Unc93B were monitored by anti-HA antibodies in the cell extracts. In all panels, blots were probed with anti-actin to control for equal loading.

(D) HA-tagged human iRhom1 and mouse iRhom2, but not Unc93B, reduced secretion and intracellular levels of Flag-EGF-A1031F.

(E) iRhom1 and iRhom2 destabilize myc-EGF levels in cell extracts, but a KDEL-tagged (and therefore ER-localized) catalytically inactive mutant of mouse RHBDL2 (HA-R2SA-KDEL) has no effect.

See also Figure S3.

Second, once Gurken has been solubilized by an active rhomboid, it is no longer subject to iRhom-mediated destabilization. Third, the fact that ER-restricted Rhomboid-1 rescues much more efficiently than Golgi-localized Rhomboid-1 argues that iRhom function occurs in the ER, not later in the secretory pathway. The slight rescue by wild-type Rhomboid-1 presumably reflects the low steady-state level in the ER as it is trafficked to the Golgi apparatus.

iRhom Acts in Absence of Active Rhomboid

Two classes of mechanism could explain our data: the iRhoms might either inhibit the function of active rhomboid proteases, for example by a dominant-negative effect, or could act directly on substrates, destabilizing them and preventing them from being cleaved by proteases. We took advantage of the absence of endogenous rhomboid activity in COS7 cells (Urban and Freeman, 2003; Adrain et al., 2011) to distinguish these models by testing the effect of iRhom in the absence of an active rhomboid. Human iRhom1 or mouse iRhom2 were coexpressed with EGF and other EGF family ligands. Under these conditions, ligand release was dependent on ADAM family metalloproteases, which were not chemically inhibited (as they were in some previous experiments). Strikingly, the secretion and intracellular levels of all EGFR ligands tested (EGF, TGFα, Epiregulin [EPR], Amphiregulin [AREG], Betacellulin [BTC], and Neuregulin 4 [Nrg4]) were downregulated by the expression of either iRhom (Figures 6A and 6B and Figures S3A–S3D). Two control proteins, prolactin, a constitutively secreted protein, and Delta, a membrane protein that is subject to metalloprotease shedding, but not cleaved by rhomboids, were unaffected by iRhom expression (Figure 6C and Figure S3E). As before, specificity was demonstrated by showing that Unc93B did not cause the same effect (Figures 6A–6C and Figures S3A–S3D). By repeating the experiment in HeLa cells, we also confirmed that the specific downregulation of EGF by iRhoms was not limited to COS cells (Figure S3F). These experiments clearly demonstrate that iRhoms can downregulate mammalian EGF family ligands (not all of which are rhomboid substrates [Adrain et al., 2011]) in the absence of any active rhomboid, thereby strongly supporting a model whereby iRhoms act directly on EGFR ligands (or possibly other clients), rather than by inhibiting the rhomboid enzymes themselves.

We further tested this model by examining whether a mutant version of EGF (A1031F) that cannot be cleaved by rhomboids but is susceptible to ADAM release (Adrain et al., 2011) was sensitive to iRhom coexpression. Both human iRhom1 and mouse iRhom2 downregulated EGF^{A1031F} and inhibited its release into the medium of COS7 cells (Figure 6D). This further

confirms that iRhoms can act on proteins that are not themselves substrates of rhomboid proteases.

Is the iRhom effect simply a consequence of an interaction between a catalytically dead rhomboid and a potential substrate? We tested this by investigating whether catalytic mutants of mouse RHBDL2 would mimic the iRhom effect on EGF. No downregulation of EGF was detected upon expression of an ER-targeted mutant of RHBDL2 (Figure 6E).

In summary, these experiments lead us to conclude that *Drosophila* and mammalian iRhoms share a conserved function; that iRhoms inhibit rhomboid-catalyzed release of growth factors by acting on the potential rhomboid substrates, rather than by inhibiting the enzymes themselves; and that the iRhoms have a specific function beyond just being catalytically inactive rhomboid proteases. An important further conclusion is that, although iRhoms show significant specificity, they can act on proteins that are not direct rhomboid substrates.

iRhoms Trigger Proteasomal Degradation of Rhomboid Substrates

The observed reduction of EGF family ligands induced by iRhoms suggested that they might be degraded in the cell. We therefore asked whether inhibition of the proteasome would suppress iRhom-induced downregulation. Myc-tagged mouse EGF was coexpressed with iRhom1 in the presence or absence of proteasomal inhibitors. As expected, iRhom expression caused a substantial reduction of EGF, both in cell extracts and secreted into the culture medium. This reduction was completely rescued by treatment with MG132, implying that the destabilization of EGF depended on proteasomal activity (Figure 7A). This result was confirmed with another proteasome inhibitor, lactacystin (Figure S4A). We also used qPCR to show that the observed increases in EGF levels were not caused by nonspecific transcriptional effects of the proteasome inhibitors (Figures S4B and S4C). Because EGF resides in the secretory pathway, it is not directly accessible to proteasomes in the cytoplasm. However, proteins can be extracted from the ER for proteasomal destruction in a process called ER-associated degradation (ERAD) (Brodsky and Wojcikiewicz, 2009). The location of iRhoms in the ER is consistent with a potential role in promoting ERAD. Interestingly, even in the absence of iRhom, MG132 treatment also slightly increased the steady-state level of intracellular EGF (Figure 7A), implying that, even under normal conditions, some EGF is degraded by the proteasome.

The conclusion that iRhom drives EGF into the ERAD machinery suggests that the two proteins might directly interact in the ER. Indeed, Flag-tagged EGF was specifically coimmunoprecipitated by HA-tagged human iRhom1 and mouse iRhom2, whereas control proteins, TGN36 and prolactin, were not (Figure 7B).

To monitor the kinetics of intracellular EGF, which can leave the cell by secretion or be degraded by the proteasome, we performed a pulse-chase experiment (Figure 7C). Because iRhom is expressed before the label is added, the iRhom effect is already apparent at the earliest stage of the chase, complicating the interpretation of this experiment. Nevertheless, this kinetic analysis is fully consistent with the steady-state data (Figure 7A and Figure S4A). In untreated cells, a pulse of labeled EGF (Figure 7C, blue diamonds) disappears from cells during a 3 hr time course. As expected, coexpression of iRhom1 reduces the level of EGF (Figure 7C, green triangles); again, all EGF disappears during the 3 hr chase. Addition of MG132 to inhibit the proteasome increases the initial level of EGF, confirming that ERAD contributes to EGF homeostasis (Figure 7A), but most of the EGF is still secreted by the cell over 3 hr (Figure 7C, red squares). Interestingly, coexpression of iRhom in cells in which the proteasome is inhibited (Figure 7C, purple crosses) slows down EGF secretion. This partial ER-anchoring effect by iRhom when it cannot promote ERAD is consistent with its direct binding to EGF (Figure 7B).

Finally, the in vivo relevance of this model was tested by using *Drosophila* genetics to look for evidence that ERAD contributes to the normal regulation of EGFR signaling. Inhibition of EGFR signaling by overexpression of either Sprouty or Argos was suppressed by RNAi knockdown of the ERAD factors Hrd1 (Figure 7D) or EDEM2 (Figure S4D). This implies that ERAD does indeed regulate EGFR signaling in the *Drosophila* eye. Overall, these data suggest that EGF levels in *Drosophila* and perhaps in mammals are normally kept in balance by low-level ERAD and that iRhoms exploit this mechanism to regulate growth factor signaling (Figure 7E).

DISCUSSION

We report that iRhoms, which evolved from active rhomboids, act in *Drosophila* as negative regulators of rhomboid-dependent proteolysis, thereby inhibiting EGF receptor signaling in the nervous system. iRhom mutants have activity defects that are consistent with a role for EGFR signaling in controlling sleep in flies. We have shown that iRhom function, binding to EGF family ligands in the ER and allowing them to be targeted for degradation by ERAD, is shared between *Drosophila* and mammals. These results provide an unexpected and conserved mechanistic link between the cellular quality control machinery and the regulation of growth factor signaling.

ER Quality Control and the Regulation of Intramembrane Proteolysis

Intramembrane proteases are potentially dangerous enzymes: they catalyze the irreversible cleavage of proteins that trigger important cellular processes. Their regulation is therefore paramount, but little is known about how this is achieved under physiological conditions. Nevertheless, several lines of evidence suggest that intramembrane proteases rely heavily on regulated segregation of substrate and enzyme (Freeman, 2008). For example, in *Drosophila*, the type II membrane protein Star regulates access of substrates to rhomboids (Lee et al., 2001; Urban et al., 2001). Similarly, the trafficking of the site-2 protease substrates SREBP or ATF6 to the Golgi apparatus, the location of S2P, is highly regulated (Sakai and Rawson, 2001; Shen et al., 2002). Here, we report a new mechanism, the specific destabilization of substrates in the ER, ultimately preventing access to an active rhomboid. Our point is not that these diverse control strategies share common mechanisms, but that they all comply with the regulatory logic of segregating substrate and

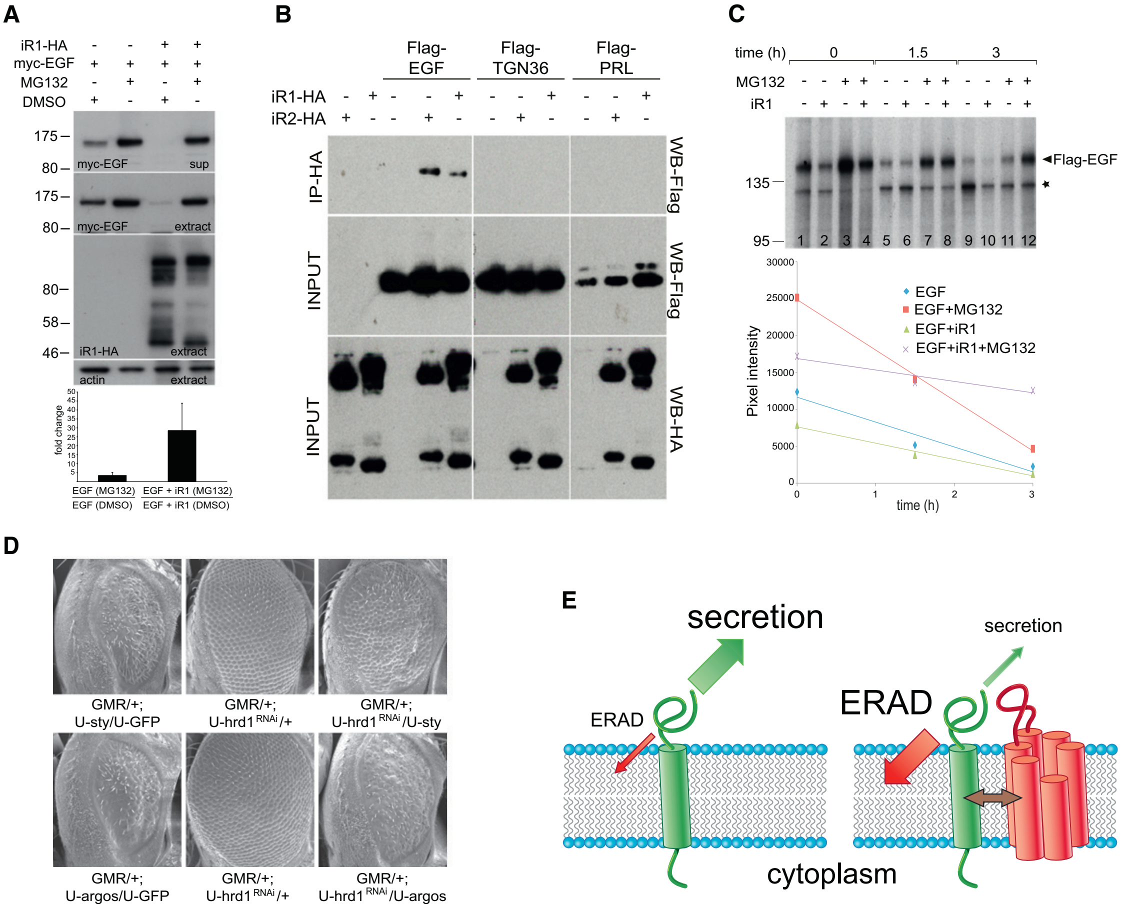

Figure 7. iRhoms Destabilize EGFR Ligands by ERAD

(A) Secretion and intracellular protein levels of myc-EGF were increased upon MG132 treatment at 10 μM for 12 hr (compare DMSO-treated lane 1 with MG132-treated lane 2 in supernatant and extracts). The inhibition of myc-EGF secretion and intracellular myc-EGF levels caused by human iRhom1-HA coexpression (compare lane 1 with lane 3) was rescued by MG132. The histogram quantifies the data: intracellular stabilization was expressed as the ratio of band intensity of MG132 treatment divided by DMSO control. MG132 stabilized steady-state levels of myc-EGF 3.4- ± 1.7-fold in the absence of coexpressed iRhom1 and 28.5- ± 15.2-fold in the presence of iRhom1. Error bars represent mean ± SD.

(B) EGF coimmunoprecipitates with both iRhom1 and iRhom2. The proteins were coexpressed in HEK293 cells as indicated. Two control proteins, TGN36 and prolactin (PRL), showed no interaction with either iRhom. The levels of each protein in the cell lysates are shown in the middle and bottom panels.

(C) A 10 min pulse of ^{35}S-methionine/cysteine was used to label Flag-tagged EGF, and its kinetics were followed for 3 hr ± MG132 and ± human iRhom1 (iR1). An autoradiogram of immunoprecipitated Flag-EGF (arrowhead) showed that iRhom1 reduced EGF levels at all chase time points (compare lanes 1/2, 5/6, and 9/10). MG132 increased EGF levels (compare lanes 1/3, 5/7, and 9/11) and rescued iRhom1-induced EGF destabilization (compare lanes 2/4, 6/8, and 10/12). The star indicates an unspecific degradation product. Quantification of the autoradiogram showed that, when the proteasome is inhibited, iRhom1 reduced the rate of secretion of EGF (compare slopes of red and purple lines).

(D) Expression of EGFR inhibitors Sprouty (*GMR/+; U-sty/U-GFP*) or Argos (*GMR/+; U-argos/U-GFP*) caused a rough eye. Reduction of ERAD by RNAi knockdown of Hrd1 (*GMR/+; U-hrd1^{RNAi}*) suppressed this EGFR inhibition.

(E) Model of iRhom function. EGF (in green) secretion is homeostatically regulated by ERAD; iRhoms (in red) bind to EGF, holding it in the ER and thereby enhancing ERAD and inhibiting secretion.

See also Figure S4.

enzyme. This contrasts with the regulation of soluble proteases, in which there is greater emphasis on regulating enzyme activity. This distinction reflects the greater ability to restrict and control the cellular location of membrane proteins than soluble proteins.

ERAD was first discovered as a mechanism for removing misfolded components of the T cell receptor complex (Lippincott-Schwartz et al., 1988). It is now clear that it contributes more generally to ER quality control and homeostasis (Brodsky and Wojcikiewicz, 2009). Our data extend the biological consequences of ERAD by demonstrating that it has been recruited in metazoans as a way of regulating intercellular signaling. Although many ERAD components have been identified, the molecular details of how proteins are targeted for recognition and retrotranslocation remain unclear (Brodsky and Wojcikiewicz, 2009). iRhoms are polytopic ER membrane proteins that can bind to at least two rhomboid substrates (this work and Nakagawa et al., 2005). They could enhance ERAD actively by introducing clients into the retrotranslocation machinery or

passively by prolonging ER retention, thereby increasing the probability of exposure to ERAD. We do not know how many proteins iRhoms affect; our genetic and cell biological data show significant specificity, but the fact that mammalian iRhoms target EGF ligands that are not rhomboid substrates (see Figure 6 and Figure S4) implies that they have evolved additional clients.

Regulation of *Drosophila* EGF Receptor Signaling

Our data also demonstrate the physiological significance of iRhoms in *Drosophila*: they are specific regulators of EGFR signaling. In mammals, our data also show that iRhoms can inhibit secretion of EGF family ligands, but in contrast to flies, the physiological significance is not yet clear. The *Drosophila* EGF receptor is probably the most genetically well-characterized growth factor receptor in any system (Shilo, 2003), and it is striking how many distinct proteins contribute to its control. Our experiments demonstrate that iRhom is a new type of regulator of EGFR activity—the most upstream control element in the pathway yet discovered. A theme that has emerged is the importance of negative feedback as a way of limiting the extent and/or amplitude of signaling (Freeman, 2000). The fact that iRhom is transcriptionally activated in the developing eye imaginal disc (Figure 2B) in cells with active EGFR signaling implies that, at least in that context, iRhom could also participate in a feedback loop. Intriguingly, its expression pattern shows that, in the eye at least, its expression peaks at a time when EGFR signaling needs to be switched off (Freeman, 1996). Degradation of EGFR ligands could therefore represent a robust way of preventing inappropriate EGFR signaling.

Although we can detect the role of iRhom in the development of the wing and eye under genetically sensitized conditions, the only prominent phenotype caused by loss of *Drosophila* iRhom is behavioral. This is consistent with its expression pattern, which is largely restricted to the nervous system. An important open question is whether the activity phenotype of iRhom mutant flies is a consequence of a developmental or physiological defect. Loss of iRhom causes flies to undergo excessive periods of inactivity during the daytime, when wild-type flies are active. In fact, iRhom mutants appear to be the first loss-of-function mutations to have an excess sleep-like phenotype (Cirelli and Bushey, 2008). *Drosophila* has become a genetic model to investigate the neuronal and molecular mechanisms of sleep (Greenspan et al., 2001), and our data support the recent report that abnormally high EGFR activity in the CNS leads to inactivity in flies (Foltenyi et al., 2007).

Evolutionary Significance of iRhoms

The iRhoms have evolved from rhomboid proteases that lost their catalytic activity but retained their location in the secretory pathway and the ability to bind their substrates. This allowed them to acquire new functions as specific regulators of secreted proteins, without retaining a direct mechanistic interaction with the proteases. Because many mutations will lead to loss of catalytic activity, the evolution of regulatory proteins from "dead" enzymes might be quite common. The expression pattern, cellular location, and substrate binding capacity of such proteins provide an ideal platform for the subsequent acquisition of specific regulatory properties. Consistent with this idea is the striking existence of inactive cognates of most proteases—indeed of many enzymes of all families (Pils and Schultz, 2004; Rawlings et al., 2010). Little or nothing is known about most of these pseudoenzymes, the widespread existence of which has only become apparent as genomes have been extensively sequenced. But we find it an attractive idea that many will have regulatory functions related to the enzymes from which they evolved.

EXPERIMENTAL PROCEDURES

Drosophila Strains and Genetics

All crosses were performed at 25°C unless otherwise stated. Fly strains are described in the Supplemental Information.

Generation of iRhom Knockout Flies

The $iRhom^{KO1/KO1}$ flies were generated by ends-out homologous recombination (Gong and Golic, 2003). The procedure is described in the Supplemental Information.

In Situ Hybridization

The 2.4 kb *iRhom* coding sequence was cloned into pENTR/SD/D-TOPO vector (Invitrogen) using directional TOPO cloning according to manufacturer's instructions. DIG-labeled RNA (Roche) antisense probes were transcribed using the T7 promoter. In situ hybridization on imaginal discs and embryos were carried out using standard procedures (Cubas et al., 1991).

Scanning Electron Microscopy of Adult Eyes

Flies were frozen for at least 1 hr at −80°C, mounted onto aluminum electron microscope specimen stubs, and coated with 20 nm of a gold-palladium mixture. Samples were viewed on a Philips XL30 scanning electron microscope.

Cloning and Point Mutagenesis

Plasmids were generated according to standard cloning techniques, and their detailed description is given in the Supplemental Information.

Protein Expression, Purification, and Rhomboid In Vitro Assays

Providencial stuartii rhomboid AarA was expressed in *E. coli* and purified according to published protocols (Stevenson et al., 2007). In vitro activity assays were carried out as described (Strisovsky et al., 2009).

Immunofluorescence

COS7 cells were stained as described in Lee et al. (2001), and imaging was performed using a Zeiss 710 Confocal Microscope. Primary antibodies were mouse monoclonal anti-HA 16B12 (1:100, Covance), mouse monoclonal anti-P230 (1:100, BD Bioscience), and polyclonal anti-PDI (1:250, Calbiochem). Alexa Fluor 568 (red)- and Alexa Fluor 488 (green)-conjugated secondary antibodies from Molecular Probes were used at 1:500.

Cell Culture-Based Assays

Cells were grown under standard conditions. Assay methods and antibody details are described in the Supplemental Information.

Quantitative RT-PCR Measurements of EGF mRNA Levels

Assay methods and reagents are described in the Supplemental Information.

Activity Data Collection, Fly Tracking, Velocity Calculations, and Statistical Analysis

Locomotor activity was monitored using the DAMS/Trikinetics system (Joiner et al., 2006), and sleep was measured in 5 min bins as previously described (Shaw et al., 2000). Waking activity (counts per minute) was calculated by averaging activity counts for every active 1 min period (Andretic and Shaw, 2005). All flies were kept on a 12 hr light/dark cycle at 25°C. Individual flies were filmed

with a video camera at 25 frames per second. Flies were tracked manually using the ImageJ plug-in Manual Tracking (http://rsb.info.nih.gov/ij/plugins/index.html).

SUPPLEMENTAL INFORMATION

Supplemental Information includes Extended Experimental Procedures, four figures, one table, and one movie and can be found with this article online at doi:10.1016/j.cell.2011.02.047.

ACKNOWLEDGMENTS

We thank Yonka Christova, Marius Lemberg, Marc de la Roche, Amita Sehgal, Sean Munro, Don Ryoo, and Simon Bullock for reagents, discussion, and unpublished data. M.Z. was supported by an Erwin-Schrödinger fellowship from the Austrian FWF and an APART fellowship from the Austrian Academy of Science. C.A. was supported by a long-term fellowship from The International Human Frontier Science Program Organization and was the recipient of an EMBO Long-Term Fellowship. K.S. acknowledges support from Marie Curie Intraeuropean Fellowship and an EMBO Long-Term Fellowship.

Received: May 26, 2010
Revised: November 26, 2010
Accepted: February 28, 2011
Published online: March 24, 2011

REFERENCES

Adrain, C., Strisovsky, K., Zettl, M., Hu, L., Lemberg, M.K., and Freeman, M. (2011). Mammalian EGF receptor activation by the rhomboid protease RHBDL2. EMBO Rep., in press. 10.1038/embor.2011.50.

Andretic, R., and Shaw, P.J. (2005). Essentials of sleep recordings in Drosophila: moving beyond sleep time. Methods Enzymol. *393*, 759–772.

Bartlett, G.J., Borkakoti, N., and Thornton, J.M. (2003). Catalysing new reactions during evolution: economy of residues and mechanism. J. Mol. Biol. *331*, 829–860.

Blobel, C.P., Carpenter, G., and Freeman, M. (2009). The role of protease activity in ErbB biology. Exp. Cell Res. *315*, 671–682.

Brinkmann, M.M., Spooner, E., Hoebe, K., Beutler, B., Ploegh, H.L., and Kim, Y.M. (2007). The interaction between the ER membrane protein UNC93B and TLR3, 7, and 9 is crucial for TLR signaling. J. Cell Biol. *177*, 265–275.

Brodsky, J.L., and Wojcikiewicz, R.J. (2009). Substrate-specific mediators of ER associated degradation (ERAD). Curr. Opin. Cell Biol. *21*, 516–521.

Brown, M.S., and Goldstein, J.L. (1997). The SREBP pathway: regulation of cholesterol metabolism by proteolysis of a membrane-bound transcription factor. Cell *89*, 331–340.

Cirelli, C., and Bushey, D. (2008). Sleep and wakefulness in Drosophila melanogaster. Ann. N Y Acad. Sci. *1129*, 323–329.

Cubas, P., de Celis, J.F., Campuzano, S., and Modolell, J. (1991). Proneural clusters of achaete-scute expression and the generation of sensory organs in the Drosophila imaginal wing disc. Genes Dev. *5*, 996–1008.

Foltenyi, K., Greenspan, R.J., and Newport, J.W. (2007). Activation of EGFR and ERK by rhomboid signaling regulates the consolidation and maintenance of sleep in Drosophila. Nat. Neurosci. *10*, 1160–1167.

Freeman, M. (1996). Reiterative use of the EGF receptor triggers differentiation of all cell types in the Drosophila eye. Cell *87*, 651–660.

Freeman, M. (2000). Feedback control of intercellular signalling in development. Nature *408*, 313–319.

Freeman, M. (2008). Rhomboid proteases and their biological functions. Annu. Rev. Genet. *42*, 191–210.

Gong, W.J., and Golic, K.G. (2003). Ends-out, or replacement, gene targeting in Drosophila. Proc. Natl. Acad. Sci. USA *100*, 2556–2561.

Greenspan, R.J., Tononi, G., Cirelli, C., and Shaw, P.J. (2001). Sleep and the fruit fly. Trends Neurosci. *24*, 142–145.

Haass, C., and De Strooper, B. (1999). The presenilins in Alzheimer's disease—proteolysis holds the key. Science *286*, 916–919.

Joiner, W.J., Crocker, A., White, B.H., and Sehgal, A. (2006). Sleep in Drosophila is regulated by adult mushroom bodies. Nature *441*, 757–760.

Lee, J.R., Urban, S., Garvey, C.F., and Freeman, M. (2001). Regulated intracellular ligand transport and proteolysis control EGF signal activation in Drosophila. Cell *107*, 161–171.

Lemberg, M.K., and Freeman, M. (2007). Functional and evolutionary implications of enhanced genomic analysis of rhomboid intramembrane proteases. Genome Res. *17*, 1634–1646.

Lippincott-Schwartz, J., Bonifacino, J.S., Yuan, L.C., and Klausner, R.D. (1988). Degradation from the endoplasmic reticulum: disposing of newly synthesized proteins. Cell *54*, 209–220.

Makinoshima, H., and Glickman, M.S. (2006). Site-2 proteases in prokaryotes: regulated intramembrane proteolysis expands to microbial pathogenesis. Microbes Infect. *8*, 1882–1888.

McQuibban, G.A., Saurya, S., and Freeman, M. (2003). Mitochondrial membrane remodelling regulated by a conserved rhomboid protease. Nature *423*, 537–541.

Nakagawa, T., Guichard, A., Castro, C.P., Xiao, Y., Rizen, M., Zhang, H.Z., Hu, D., Bang, A., Helms, J., Bier, E., and Derynck, R. (2005). Characterization of a human rhomboid homolog, p100hRho/RHBDF1, which interacts with TGF-alpha family ligands. Dev. Dyn. *233*, 1315–1331.

Pils, B., and Schultz, J. (2004). Inactive enzyme-homologues find new function in regulatory processes. J. Mol. Biol. *340*, 399–404.

Rawlings, N.D., Barrett, A.J., and Bateman, A. (2010). MEROPS: the peptidase database. Nucleic Acids Res. *38* (Database issue), D227–D233.

Sakai, J., and Rawson, R.B. (2001). The sterol regulatory element-binding protein pathway: control of lipid homeostasis through regulated intracellular transport. Curr. Opin. Lipidol. *12*, 261–266.

Shaw, P.J., Cirelli, C., Greenspan, R.J., and Tononi, G. (2000). Correlates of sleep and waking in Drosophila melanogaster. Science *287*, 1834–1837.

Shen, J., Chen, X., Hendershot, L., and Prywes, R. (2002). ER stress regulation of ATF6 localization by dissociation of BiP/GRP78 binding and unmasking of Golgi localization signals. Dev. Cell *3*, 99–111.

Shilo, B.Z. (2003). Signaling by the Drosophila epidermal growth factor receptor pathway during development. Exp. Cell Res. *284*, 140–149.

Stevenson, L.G., Strisovsky, K., Clemmer, K.M., Bhatt, S., Freeman, M., and Rather, P.N. (2007). Rhomboid protease AarA mediates quorum-sensing in Providencia stuartii by activating TatA of the twin-arginine translocase. Proc. Natl. Acad. Sci. USA *104*, 1003–1008.

Strisovsky, K., Sharpe, H.J., and Freeman, M. (2009). Sequence-specific intramembrane proteolysis: identification of a recognition motif in rhomboid substrates. Mol. Cell *36*, 1048–1059.

Urban, S. (2009). Making the cut: central roles of intramembrane proteolysis in pathogenic microorganisms. Nat. Rev. Microbiol. *7*, 411–423.

Urban, S., and Freeman, M. (2003). Substrate specificity of rhomboid intramembrane proteases is governed by helix-breaking residues in the substrate transmembrane domain. Mol. Cell *11*, 1425–1434.

Urban, S., Lee, J.R., and Freeman, M. (2001). Drosophila rhomboid-1 defines a family of putative intramembrane serine proteases. Cell *107*, 173–182.

Urban, S., Lee, J.R., and Freeman, M. (2002a). A family of Rhomboid intramembrane proteases activates all Drosophila membrane-tethered EGF ligands. EMBO J. *21*, 4277–4286.

Urban, S., Schlieper, D., and Freeman, M. (2002b). Conservation of intramembrane proteolytic activity and substrate specificity in prokaryotic and eukaryotic rhomboids. Curr. Biol. *12*, 1507–1512.

Wang, Y., Zhang, Y., and Ha, Y. (2006). Crystal structure of a rhomboid family intramembrane protease. Nature *444*, 179–180.

Wasserman, J.D., Urban, S., and Freeman, M. (2000). A family of rhomboid-like genes: Drosophila rhomboid-1 and roughoid/rhomboid-3 cooperate to activate EGF receptor signaling. Genes Dev. *14*, 1651–1663.

Weihofen, A., Binns, K., Lemberg, M.K., Ashman, K., and Martoglio, B. (2002). Identification of signal peptide peptidase, a presenilin-type aspartic protease. Science *296*, 2215–2218.

Yan, Z., Zou, H., Tian, F., Grandis, J.R., Mixson, A.J., Lu, P.Y., and Li, L.Y. (2008). Human rhomboid family-1 gene silencing causes apoptosis or autophagy to epithelial cancer cells and inhibits xenograft tumor growth. Mol. Cancer Ther. *7*, 1355–1364.

Yogev, S., Schejter, E.D., and Shilo, B.Z. (2008). Drosophila EGFR signalling is modulated by differential compartmentalization of Rhomboid intramembrane proteases. EMBO J. *27*, 1219–1230.

Zou, H., Thomas, S.M., Yan, Z.W., Grandis, J.R., Vogt, A., and Li, L.Y. (2009). Human rhomboid family-1 gene RHBDF1 participates in GPCR-mediated transactivation of EGFR growth signals in head and neck squamous cancer cells. FASEB J. *23*, 425–432.

EXTENDED EXPERIMENTAL PROCEDURES

Drosophila Strains and Genetics

Drosophila iRhom (Q76NQ1, cDNA HL06695) was cloned into pUAST vector (pTW, The *Drosophila* Gateway vector collection, http://www.ciwemb.edu/labs/murphy/Gateway%20vectors.html) using the Gateway system (Invitrogen). *UAS-iRhom* flies were generated using standard techniques. The following fly strains, described in FlyBase (http://flybase.bio.indiana.edu/), were used: *yw*, OregonR, *w1118; Df(2L)J17*, *b[1]/CyO* (31C-31F; 2L:10280567..10557961), *w1118; P(w[+mC] = UAS-myr-mRFP)2/TM6B*, *Tb1*, *y w; P(w[+mC] = UAS-2xEGFP)AH3*, *P(ftz/lacC)4*, hh^{21}*/TM3 Sb1*, ptc^{9} *cn1 bw1 sp1/CyO*, dpp^{hr92}, *w; mad*^{12} *P(neoFRT)40A/CyO*, *sev-rho* (Freeman et al., 1992), *UAS-DNEgfr* (gift from B. Shilo), *UAS-argos* (Freeman, 1994), *UAS-sty* (Casci et al., 1999), *ELAV-Gal4* (gift from D. Crowther), *how24B-Gal4* (Chan et al., 2003), *GMR-Gal4*, $argos^{l\Delta7}$ (Freeman, 1996), sty^{S73} (Casci et al., 1999), Dl^{rev10}, *GMR-Su(H)DN*, N^{55e11}, $Hairless^{P141}$ (gifts from S. Bray); *F76e*, $pygo^{S123}$, $\Delta axin^{P}$, ΔTCF^{2}, *UAS-cadi*, *en-Gal4* (gifts from M. Bienz); *UAS-Smo5A*, *C765-Gal4* (a gift from S. Cohen), *UAS-Egfr*^{RNAi} (transformant ID 43267) and *UAS-spi*^{RNAi} (transformant ID 3920) were obtained from the Vienna *Drosophila* RNAi Center. *UAS-hrd1*^{RNAi} and *UAS-EDEM2*^{RNAi} were gifts from Don Ryoo (NYU).

Generation of iRhom Knockout Flies

The $iRhom^{KO1/KO1}$ flies were generated by ends-out homologous recombination (Gong and Golic, 2003), using the pW25 vector backbone (Gong and Golic, 2004). 3.5 kb of genomic DNA flanking the coding region of *rhomboid-5/iRhom* were cloned by recombineering (Testa et al., 2003) from a BAC (BACR30M19) containing the *rhomboid-5/iRhom* locus. All coding regions and cloning junctions were confirmed by DNA sequencing. A transgene insertion donor line on the third chromosome was used for targeting, as the *rhomboid-5/iRhom* gene is located on the second chromosome.

The targeting fragment was liberated and linearized in the female germline using FLP and I-*Sce*I, respectively, and progeny were screened for movement of the mini-white marker to the second chromosome, as well as its resistance to eyFLP (indicating that it is no longer flanked by FRT sites, as in the donor).

The successful generation of three homologous recombinants was confirmed by Southern blot and PCR with specific primers to detect the insertion of the mini-white marker and the disruption of the endogenous *iRhom* locus (Figure S1). The junctions of the resulting PCR products were sequenced to confirm site-specific integration of the targeting construct.

Cloning and Point Mutagenesis

Expression plasmids for *Drosophila* Rho-1, Rho-1 SA, KDEL-Rho1, KDEL-Rho1SA, mouse RHBDL2, RHBDL2 SA, RHBDL2 SA-KDEL, RHBDL-3 WNT3a (gift from Mariann Bienz) and mouse Unc93B with an N-terminal triple HA tag in pcDNA3.1 (Invitrogen) were described previously (Urban et al., 2001; Lohi et al., 2004; Brinkmann et al., 2007). The untagged Star expression construct was described previously (Lee et al., 2001). *Drosophila* iRhom (Q76NQ1, cDNA HL06695), human iRhom1 (NP_ 071895, Image clone 4650826) and mouse iRhom2 (AAH52182, Image clone 3910543) were cloned into pEGFP-N1 (BD Biosciences Clontech), and were C-terminally fused to a triple HA tag, replacing the EGFP cassette. Spitz (AAA28894) was cloned with a triple FLAG-tag in the N-terminus. *Drosophila* Delta, Grk and mouse TGN36 (IMAGE cDNA clone 3157708) were cloned without their signal peptides into a pcDNA3.1-based expression vector containing the Spitz signal peptide fused to a triple Flag tag at their N-terminus. Mouse Amphiregulin (NM_009704.3), mouse Betacellulin (NM_007568.3), mouse Epiregulin (NM_007950.2) and mouse EGF (NM_010113.2) were cloned without their signal peptides into a pcDNA3.1-based expression vector containing the Spitz signal peptide fused to a triple myc tag. Similarly, the following were cloned into an expression vector comprising the Spitz signal peptide fused to a triple FLAG tag: human prolactin (NM_000948.3), human HB-EGF (NM_001945.2) and mouse EGF (NM_010113.2). *Drosophila* Rho1 A216P, mouse RHBDL2 A185P, AarA A149P, EGF and Spitz point mutants were generated using the Quikchange II mutagenesis kit (Stratagene) in accordance with the manufacturer's instructions, using mouse EGF cDNA (NM_010113.2) as a template.

Cell Culture-Based Assays

Cells were maintained in Dulbecco's Modified Eagles Medium supplemented with 10% fetal calf serum. For COS7-based overexpression assays, cells were first transfected in 6-well plates using Fugene 6 transfection reagent (Roche) using 250 ng of substrate and 25 ng of rhomboid or iRhom (= 1x) expression plasmids. The total DNA amount was adjusted to 1 μg with pcDNA3.1(+) vector. Eighteen hours post-transfection, cells were washed once in PBS and the secretion assay was then performed for 30 hr in serum-free medium unless stated otherwise. The culture supernatants were collected by centrifugation at 16,000 g for 10 min, and proteins precipitated by adding TCA (12% w/v). Precipitated proteins were recovered by centrifugation, washed in acetone and solubilised in 30 μl 1x SDS PAGE sample buffer; cell extracts were lysed in 150 μl of sample buffer. 15 μl of each were then electrophoresed. Samples were typically electrophoresed on 4%–20% SDS-PAGE gels (Novagen) and analyzed by Western blotting using mouse monoclonal anti-FLAG M2 antibody conjugated to horseradish peroxidase (HRP) (1:2000, Sigma) or mouse monoclonal anti-HA 16B12 (1:2000, Covance), polyclonal anti-actin (1:20 000, Abcam), polyclonal anti-Myc A-14 polyclonal (1:2000, Santa Cruz). Secondary HRP-coupled antibodies were from Santa Cruz Biotech. HRP activity was detected by enhanced chemiluminescence (GE Healthcare). Substrate conversion of Grk and levels of EGF, TGFα and Delta were calculated from the densitometric analysis of X-ray films from Western blots or radioactive exposure using Image quant software.

For co-immunoprecipitation experiments 60%–80% confluent HEK293 ET cells were transfected with the cDNA constructs indicated in Figure 7C using polyethyleneimine (PEI, 25 kDa linear, Polysciences). 20-24 hr post-transfection cells were washed twice with ice-cold PBS and incubated on ice in Triton X-100 lysis buffer (1% Triton X-100, 150 mM NaCl, 50 mM Tris-HCl, pH 7.4) containing complete protease inhibitor cocktail (Roche). The post-nuclear supernatants were pre-cleared by incubating with mouse IgG-agarose (Sigma) for one hour at 4°C. The HA-tagged iRhoms where immunoprecipitated using anti-HA affinity gel (Sigma) for one hour at 4°C, and the beads were subsequently washed three times with cold lysis buffer containing 300 mM NaCl. The immunoprecipitaes were eluted with 1x SDS PAGE sample buffer containing 50 mM DTT, and reduced for 15 min at 37°C prior SDS-PAGE. The samples were analyzed SDS-PAGE and the FLAG-tagged co-immunoprecipitated proteins were detected by Western blotting.

For pulse chase analysis COS7 cells in 10cm plates were transfected with 1500 ng FLAG-EGF with or without 600 ng of iRhom1 and the total DNA amount was adjusted to 6 μg with pcDNA 3.1 empty vector. 24 hr post transfection, cells were pre-treated with either DMSO vehicle or 10 μM MG132 in complete DMEM/10% FCS for 3.5 hr. After this, cells were washed in PBS and then starved for 20 min in DMEM lacking cysteine and methionine containing dialyzed amino acids and supplemented with 10 mM HEPES pH 7.4 plus DMSO or MG132 as indicated above. Cells were then exposed to a precise 10 min pulse of 35S Met/Cys (a total of 240 mCi in 5 ml of medium) in the same medium, after which the radiolabel was removed. Cells were chased for the indicated time periods in complete medium supplemented with excess methionine and cysteine, containing DMSO or MG132, after which they were lysed on ice in 800 μl IP buffer (10 mM Tris pH 7.4, 150 mM NaCl, 1% TX-100 plus complete protease inhibitor cocktail [Roche]). Clarified supernatants were then pre-cleared with 30 μl mouse IgG-agarose (Sigma) for one hour at 4°C, followed by immunoprecipitation overnight with 30 μl of anti-FLAG M2-agarose (Sigma). Immunoprecipitates were then washed 3 times at room temperature in 1 ml of IP buffer containing 300 mM NaCl. Immunoprecipitates were then electrophoresed on 8% tris-glycine gels after which they were fixed, exposed for 20 min in 1.5 M sodium salicylate in 30% Methanol and dried, before exposure to photograpic film at −70°C.

Quantitative RT-PCR Measurements of EGF mRNA Levels

For assaying relative mouse EGF mRNA levels upon overexpression in the presence or absence of iRhoms ± lactacystin or MG132, RNA was extracted from COS7 cells using the RNeasy kit (QIAGEN). cDNA was then synthesized from RNA using the Superscript III kit (Invitrogen) based on 400 ng of total RNA in a 10 μl reaction. qPCR was performed on the resultant cDNA using gene expression mastermix using probes for EGF (Mm01316968_m1) and GAPDH (Hs00266705_g1) (Applied Biosystems). For comparison, the levels of EGF mRNA were normalized relative to the GAPDH levels in each sample. Subsequently, the relative amounts of EGF mRNA in the various treatments were expressed as a fraction of the treatment where EGF alone was overexpressed.

SUPPLEMENTAL REFERENCES

Brinkmann, M.M., Spooner, E., Hoebe, K., Beutler, B., Ploegh, H.L., and Kim, Y.M. (2007). The interaction between the ER membrane protein UNC93B and TLR3, 7, and 9 is crucial for TLR signaling. J. Cell Biol. *177*, 265–275.

Casci, T., Vinós, J., and Freeman, M. (1999). Sprouty, an intracellular inhibitor of Ras signaling. Cell *96*, 655–665.

Chan, Y.B., Miguel-Aliaga, I., Franks, C., Thomas, N., Trülzsch, B., Sattelle, D.B., Davies, K.E., and van den Heuvel, M. (2003). Neuromuscular defects in a Drosophila survival motor neuron gene mutant. Hum. Mol. Genet. *12*, 1367–1376.

Freeman, M. (1994). Misexpression of the Drosophila argos gene, a secreted regulator of cell determination. Development *120*, 2297–2304.

Freeman, M. (1996). Reiterative use of the EGF receptor triggers differentiation of all cell types in the Drosophila eye. Cell *87*, 651–660.

Freeman, M., and Bienz, M. (2001). EGF receptor/Rolled MAP kinase signalling protects cells against activated Armadillo in the Drosophila eye. EMBO Rep. *2*, 157–162.

Freeman, M., Kimmel, B.E., and Rubin, G.M. (1992). Identifying targets of the rough homeobox gene of Drosophila: evidence that rhomboid functions in eye development. Development *116*, 335–346.

Gong, W.J., and Golic, K.G. (2003). Ends-out, or replacement, gene targeting in Drosophila. Proc. Natl. Acad. Sci. USA *100*, 2556–2561.

Gong, W.J., and Golic, K.G. (2004). Genomic deletions of the Drosophila melanogaster Hsp70 genes. Genetics *168*, 1467–1476.

Hendricks, J.C., Finn, S.M., Panckeri, K.A., Chavkin, J., Williams, J.A., Sehgal, A., and Pack, A.I. (2000). Rest in Drosophila is a sleep-like state. Neuron *25*, 129–138.

Lee, J.R., Urban, S., Garvey, C.F., and Freeman, M. (2001). Regulated intracellular ligand transport and proteolysis control EGF signal activation in Drosophila. Cell *107*, 161–171.

Lohi, O., Urban, S., and Freeman, M. (2004). Diverse substrate recognition mechanisms for rhomboids; thrombomodulin is cleaved by Mammalian rhomboids. Curr. Biol. *14*, 236–241.

Mao, Y., and Freeman, M. (2009). Fasciclin 2, the Drosophila orthologue of neural cell-adhesion molecule, inhibits EGF receptor signalling. Development *136*, 473–481.

Shaw, P.J., Cirelli, C., Greenspan, R.J., and Tononi, G. (2000). Correlates of sleep and waking in Drosophila melanogaster. Science *287*, 1834–1837.

Testa, G., Zhang, Y., Vintersten, K., Benes, V., Pijnappel, W.W., Chambers, I., Smith, A.J., Smith, A.G., and Stewart, A.F. (2003). Engineering the mouse genome with bacterial artificial chromosomes to create multipurpose alleles. Nat. Biotechnol. *21*, 443–447.

Urban, S., Lee, J.R., and Freeman, M. (2001). Drosophila rhomboid-1 defines a family of putative intramembrane serine proteases. Cell *107*, 173–182.

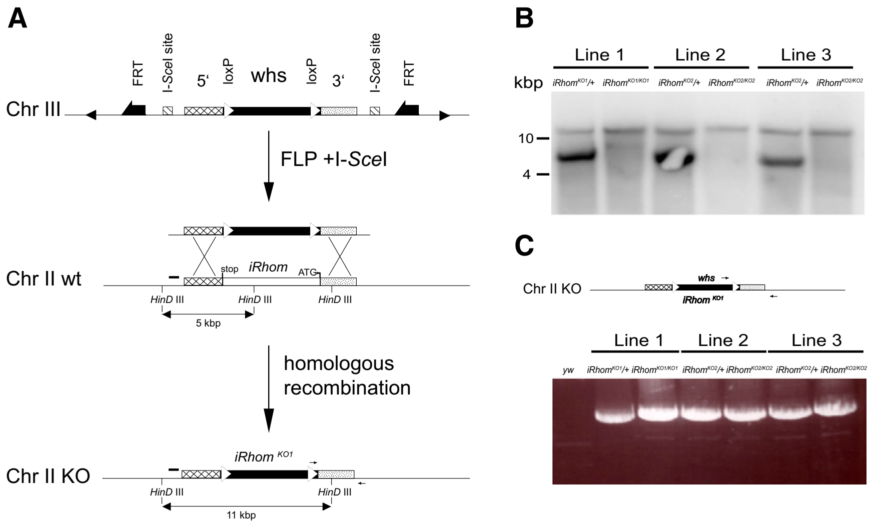

Figure S1. Generation of *iRhom/rhomboid-5* Mutant *Drosophila*, Related to Figure 2

(A) At the top is the donor construct, as it would appear on the third chromosome (Chr III) when initially introduced by P-element transformation. *FRT* sites allow the FLP recombinase to free the DNA from its chromosomal integration site. This results in circular extra-chromosomal donor DNA, which is subsequently linearized by I-*Sce*I. Homologous recombination can then occur between the donor construct comprising 5′ and 3′ homology arms either side of the mini-white marker gene (*whs*), which is flanked by *loxP* sites. Note that donor transformant lines carry the donor construct on the third chromosome and the *iRhom/rhomboid-5* locus is on the second chromosome. Upon successful homologous recombination, the *whs* marker becomes genetically linked to the second chromosome, and the donor construct loses its *FRT* sites, which was exploited to enrich for correct targeting events as described in Experimental Procedures. The lower panels show the recombination event between the extra-chromosomal donor DNA (after FLP- mediated excision and I-*Sce*I cutting) with the genomic locus containing *iRhom/rhomboid-5*. The black bar indicates the position of the DNA probe used to screen for correct site-specific integration by Southern blot. Note that the probe was generated against genomic sequence just outside the 5′ homology arm, to detect the wild-type 5kb genomic fragment after *Hin*DIII digest upon site specific integration. Correct targeting replaces the *iRhom* genomic region with the *whs* marker, leading to the loss of a *Hin*DIII site, which increases the predicted size of the genomic *Hin*DIII DNA fragment to 11kb.

(B) Southern blot analyzing three independent mutant fly lines (*KO1*, *KO2* and *KO3*) with the mini-white marker on chromosome II. All three homozygous mutant lines (*KO1-3/KO1-3*) showed the expected 11kb fragment predicted by the correct targeting, while a *Hin*DIII digest of genomic DNA from heterozygous (+/*KO1-3*) lines resulted in a 5kb wild-type and 11kb mutant fragment.

(C) Genomic PCR strategy detecting a correct genomic targeting replacing the *iRhom/rhomboid-5* locus with the mini-white gene. Note one primer was designed outside the 3′ homology arm, and the second within the mini-white gene; a PCR product can only be formed when correct targeting occurs. Correct integration was further confirmed by sequencing the PCR product covering the insertion region.

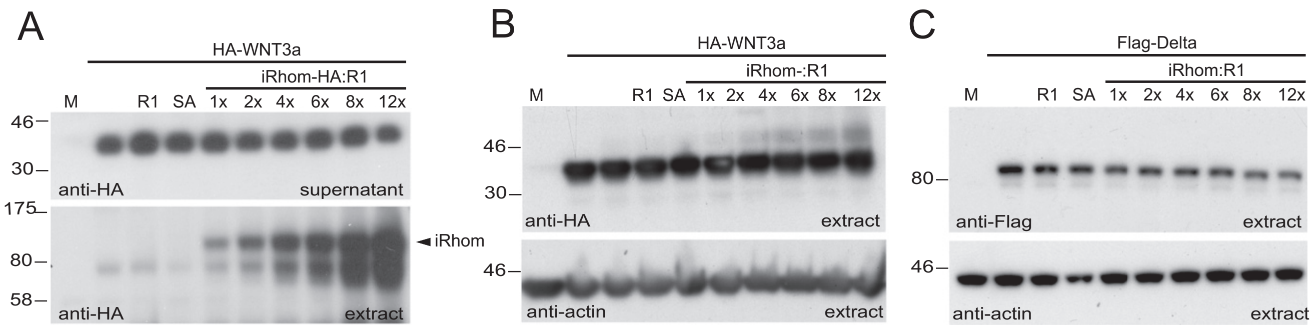

Figure S2. iRhom Did Not Have an Impact on WNT3a Secretion Intracellular WNT3a or Delta Levels, Related to Figure 5

(A) Secretion of the HA-tagged WNT3a was detected by anti-HA antibody in the supernatants (upper panel). The iRhom to *Drosophila* Rhomboid-1 (R1) ratios (iRhom:R1) indicate amounts of transfected DNA. Increasing intracellular levels of HA-tagged iRhom was confirmed by anti-HA probing of the extracts (lower panel).

(B and C) Intracellular levels of HA-tagged mouse WNT3a and *Drosophila* Flag-tagged Delta were unaffected by elevated iRhom levels (upper panel). Equal loading was confirmed by probing the cell extracts with anti-actin antibody (lower panel).

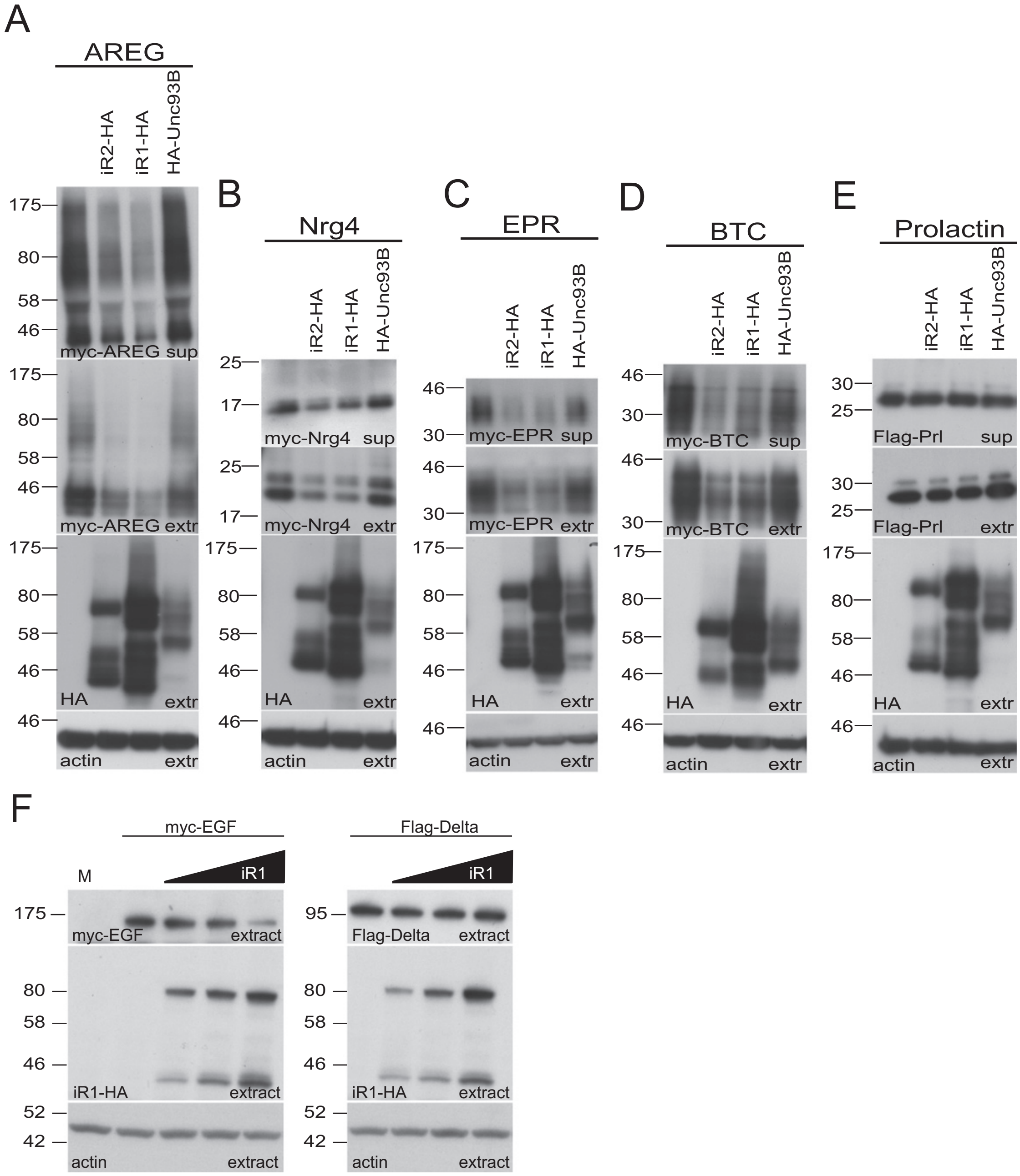

Figure S3. iRhoms Reduce Secretion and Intracellular Levels of Multiple EFGR Ligands, Related to Figure 6

(A–E) Secretion (sup, upper panel) and intracellular levels (extr, second panel from top) of the indicated EGFR ligands were decreased by human iRhom1 (iR1-HA) and mouse iRhom2 (iR2-HA) but unaffected by mouse Unc93B. Secretion and intracellular levels of Flag-tagged prolactin was unaffected by iR1, iR2 and Unc93B (E). EGFR ligands, and prolactin were detected with myc and Flag antibodies, respectively. Levels of iRhom1, iRhom2 and Unc93B were monitored by anti-HA antibodies in the cell extracts (extr, third panel from top). In all panels blots were probed with anti-actin to control for equal loading (extr, lower panel).

(F) In HeLa cells intracellular levels of myc-tagged EGF was decreased by human iRhom1 (iR1-HA) whereas levels of Flag-tagged Delta were unaffected by iR1-HA. EGF, and Delta were detected with myc and Flag antibodies, respectively. Levels of iR1 were monitored by anti-HA antibodies in the cell extracts. In all panels, blots were probed with anti-actin to control for equal loading.

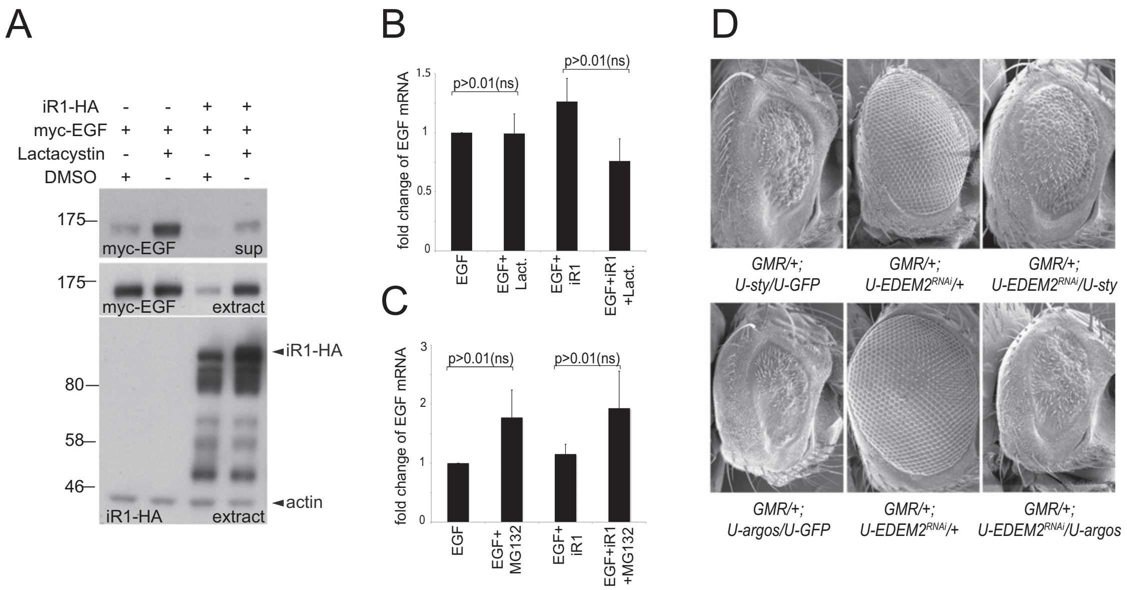

Figure S4. iRhoms Destabilize EGFR Ligands by ERAD, Related to Figure 7

(A) Secretion of myc-EGF was increased upon lactacystin treatment at 10 μM for 12 hr (compare DMSO treated lane 1 with lactacystin treated lane 2 in supernatant and extracts). The inhibition of myc-EGF secretion and intracellular myc-EGF levels caused by human iRhom1-HA coexpression (compare lane 1 with lane 3) was rescued by lactacystin. Probing cell extracts with anti-HA antibody controls for iRhom1 and actin levels.

(B and C) EGF mRNA levels in the presence or absence of human iRhom1 (iR1) ± lactacystin or MG132 were measured by real time q-PCR. Inputs were normalized to endogenously expressed GAPDH and results graphed as relative fold changes of EGF mRNA levels. The data represents four independent experiments. In both cases the observed changes were not statistically significant (ns), as determined by Student's t test. Error bars represent mean ± SD.

(D) Expression of EGFR inhibitors Sprouty (*GMR/+; U-sty/U-GFP)* or Argos (*GMR/+; U-argos/U-GFP*) caused a rough eye. ERAD inhibition in the eye on (*GMR/+; U-EDEM2^{RNAi}*) effectively suppressed EGFR inhibition mediated by Sprouty (*GMR/+; U-sty/U-EDEM2^{RNAi}*) and Argos (*GMR/+; U-argos/U- EDEM2^{RNAi}).*
